# Review of Hollow Fiber Membranes for Gas Separation: Exploring Fundamentals and Recent Advancements

**DOI:** 10.3390/membranes15080246

**Published:** 2025-08-11

**Authors:** Valentina Grosso, Carmen Rizzuto, Elena Tocci, Alessio Fuoco, Mariagiulia Longo, Marcello Monteleone, Pegah Hajivand, Johannes C. Jansen, Elisa Esposito

**Affiliations:** Institute on Membrane Technology, CNR-ITM, Via P. Bucci 17/C, 87036 Rende, CS, Italy; v.grosso@itm.cnr.it (V.G.); c.rizzuto@itm.cnr.it (C.R.); e.tocci@itm.cnr.it (E.T.); m.longo@itm.cnr.it (M.L.); m.monteleone@itm.cnr.it (M.M.); p.hajivand@itm.cnr.it (P.H.); johannescarolus.jansen@cnr.it (J.C.J.)

**Keywords:** hollow fiber membrane, membrane material, application of hollow fibers, gas separation

## Abstract

Hollow fiber membranes have revolutionized various gas separation processes due to their unique characteristics such as high surface area, small system footprint, and high energy efficiency compared to flat sheet or spiral wound membranes. This review analyzes the current state of the art of hollow fiber technology, exploring its diverse applications across various fields. Over the past ten years, research has primarily focused on improving hollow fiber fabrication techniques, including phase inversion, electrospinning, and 3D printing, highlighting their impact on membrane performance and selectivity. Furthermore, we discuss the challenges and future perspectives of hollow fiber technology, focusing on the development of novel materials and surface modifications to enhance membrane durability and efficiency. Finally, this review provides an overview of current gas separation techniques, spanning both conventional and next-generation methods, based on the foreseen field of exploitation of hollow fiber membranes.

## 1. Introduction

**Figure 1:**
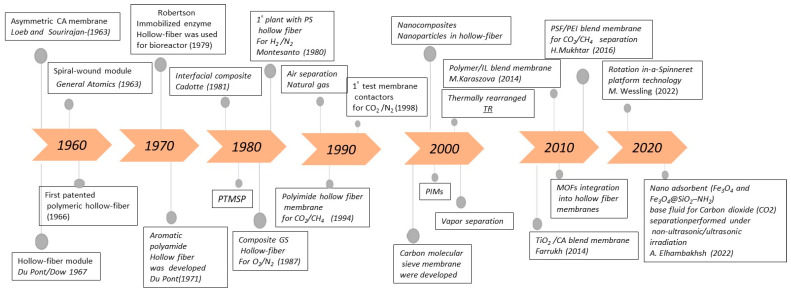
Main events marking the historical development of membranes for gas separation.

Gas separation is a crucial unit operation that is frequently applied in several sectors, including natural gas processing, biogas upgrading, emissions control, hydrogen production, and chemical purification [1]. Figure 1 reports the main events marking the historical development of membranes for gas separation. In the 1850s, the pioneering work of Thomas Graham on gas diffusion through polymeric films [2] laid the foundation for gas separation using membranes with the development of Graham’s law. In the early 1920s, Lord Rayleigh conducted experiments to measure the permeability of oxygen, nitrogen, and argon through rubber [3]. A significant breakthrough occurred in the 1960s [4] with the invention of asymmetric polymeric membranes by Loeb and Sourirajan, which enabled larger-scale gas separation processes. Later the first patented polymeric hollow fiber was introduced by Mahon in the 1966s [5]. To address surface defects in asymmetric membranes, Henis and Tripodi [6] developed a coating technique using silicone rubber. Monsanto commercialized the first gas separation membrane, Prism^®^, for hydrogen separation in 1980 [2,6,7,8,9]. In the present, membrane technology is applicable in a variety of gas separation processes, including carbon dioxide (CO_2_) capture [10], CO_2_/methane (CH_4_) separation [11], hydrogen (H_2_) purification [12], nitrogen (N_2_) production [13], and oxygen (O_2_) enrichment [14].

Polyethersulfone (PES) [15], polyphenylsulfone (PSF) [16], polyetherimide (PEI) [17], polyethylenimine (PEI) [18], polyvinylidene fluoride (PVDF) [19], polyvinyl chloride (PVC) [20], poly(1-trimethylsilyl-1-propyne) (PTMS) [21] and cellulose acetate (CA) [22] are among the most popular polymers used in gas separation membranes. Hollow fiber membranes (HFMs) provide advantages such as self-supported, compact design, and a higher surface area-to-volume ratio compared to flat sheet membranes in spiral wound or plate and frame modules. This is due to the small fiber diameter, allowing more membrane surface area to be packed into a given volume [23]. The development of hollow fiber membranes began in 1966 with Dow Chemical, followed by commercialization by companies such as Monsanto and DuPont [24]. 

The material properties of membranes play a crucial role in gas separation, which occurs at the molecular level through the interaction between the permeating gases and the polymer matrix. A comprehensive understanding of the required and potential advancements in membrane materials is essential from both a materials science and process economics perspective [23]. 

The Robeson diagram, introduced in 1991, established a benchmark for comparing the performance of new materials in gas separation, based on their permeability–selectivity trade-off [25]. Freeman later explored the theoretical underpinnings of this trade-off and analyzed the individual contributions of solubility and diffusion to the upper bound [26]. Researchers continue to seek innovative materials that can push the upper bound towards higher permeability and selectivity [23]. However, these innovative materials may also have significant disadvantages, such as high production costs and difficulties in scaling up their manufacturing. Thus, to enhance the performance of existing membranes, the easiest approach is to modify their surface in order to increase the selectivity performance.

Furthermore, hollow fiber units are vulnerable to clogging and fouling caused by tiny particles. This is because the fibers are closely packed, leaving little space between them, and the fibers themselves are very narrow. As a result, the membranes’ ability to separate substances and their overall lifespan are negatively impacted. This can lead to increased costs for cleaning the membranes [24]. There are different strategies to overcome these limitations by membrane modification. Typical strategies include the following: modifying membrane surface characteristics by blending with secondary polymers, such as polyvinylpyrrolidone (PVP) [27] and polyethylene glycol (PEG) [28]; inclusion of inorganic nanofillers such as zeolite and silica [16,29]; atom transfer radical polymerization (ATRP) [30]; surface segregation [31]; UV-assisted graft polymerization [32]; plasma-induced graft polymerization [33]; co-extrusion fabrication [34,35]; and surface coating [36,37].

Thin-film composite hollow fiber membranes (TFC-HFMs) consist of a very thin, dense selective layer coated on a porous support made from different materials. The porous substrate is typically fabricated using a phase inversion method, while the dense selective layer is prepared through plasma polymerization [38], dip coating [39], dual-layer spinning [40], or interfacial polymerization [41].

The membrane preparation step is the second most critical factor in creating the next generation of polymeric HF gas separation membranes, following only the selection of materials with superior gas transport properties. Lastly, a thorough understanding of the membrane’s properties under operating conditions is essential, and this includes how these properties change over time with temperature variations, different feed gas compositions, and in the presence of trace contaminants or humidity [23].

This review on hollow fiber membranes highlights how hollow fiber technology addresses emerging challenges, effectively closing existing gaps in current industrial gas separation processes. Moreover, the past decade has seen a remarkable rise in advanced materials specifically designed for gas separation, with superior properties as well as in green materials. This review thoroughly examines these developments, providing a comprehensive comparative analysis. We compared their advantages and disadvantages in terms of both their preparation methods and their gas transport performance when prepared into hollow fiber systems for gas separation. Furthermore, we analyzed the indispensable role of computational tools in modern materials science and membrane engineering. Our review details how these tools are important for predicting hollow fiber gas separation performance and, in many cases, explaining the behavior of these systems.

Finally, by presenting and analyzing various processes that use hollow fibers for gas separation, we offer critical insights into their commercialization potential.

## 2. Material for HFMs

Nowadays, HFMs are a consolidated technology in different industrial gas separation sectors, and depending on the application, HFMs differ from each other in their morphology, structure, and material. According to the separation needs, it is necessary to choose among a variety of HFM forming materials, which must be pressure-resistant, temperature-resistant, and/or plasticization-resistant in the expected operational conditions. The selection of the right polymeric membrane material for a given gas separation generally depends most on its permeability and selectivity, which are intrinsic properties of the material. As it is well known, glassy and rubbery polymers suffer from a trade-off between permeability and selectivity, and this is well represented by the original 1991 Robeson curve and its following updates up to 2019 [25,42,43,44]. In general, glassy polymers are highly selective and less permeable, whereas rubbery polymers are highly permeable and less selective.

### 2.1. Rubbery Materials

These materials operate above their glass transition temperature, a condition in which rubbery materials present a high chain mobility that facilitates gas diffusion. Therefore, they are generally more permeable than glassy polymers, while their separation performance relies largely on solubility selectivity because not having rigid polymer-chains, which determine a free volume with well-defined space, they cannot exploit the size-selective transport. The flexibility of rubbery membranes does not allow for the preparation of self-supported HFMs of Loeb–Sourirajan type. Therefore, rubbery HF membranes of poly(ether block amide) [45], silicone [46], or a blend based on polymer/ionic liquid [47] are prepared in the form of dual-layer composite hollow fibers, with a thin dense layer of rubber on a porous support made with a different material suitable to provide mechanical resistance [48,49]. The dominant role of solubility selectivity in the separation based on the solution-diffusion mechanism, makes rubbery HFMs more appropriate and good candidates, especially for separation the highly condensable gases or vapors, and for applications such as removal of volatile organic compounds (VOCs) from air.

### 2.2. Glassy Materials

These materials operate below their glass transition temperature, where the motion of the polymer chains is frozen in a highly rigid state. The chains are generally much stiffer than those of the rubbery polymers. Therefore, they are generally more selective than rubbery polymers, and their separation performance relies largely on diffusion selectivity thanks to their rigidity, needed for size-selective transport. With the exception of a few special classes, they are characterized by a low fractional free volume and the absence of limited interconnections between voids. Exceptions are the glassy perfluoropolymers (PFPs), which have a stiff polymer backbone but present a high fractional free volume due to their low cohesive forces, and the so-called polymers of intrinsic microporosity (PIMs) in which the free volume is largely interconnected for the transport of small gases.

The material is responsible for the gas transport properties, but it even determines the hollow fiber membranes preparation method. Glassy polymers allow us to prepare HFMs self-supported as Loeb–Sourirajan type and even dual- or multi-layer composite HFMs. Different types of glassy polymers have been studied for the preparation of hollow fiber membranes; however, commercial membranes are based on relatively few types of polymers. Some of the most common polymers studied as HF gas separation membranes are PSf [50], Polyetherimide (PEI) [51], PES [52], and Polyimide (PI) [53]. These polymers, being soluble in a wide range of organic solvents, can be produced by the NIPS method.

The relatively new class of PIMs has received a lot of attention as gas separation membrane materials since their introduction in 2004 [54]. Due to their high cost and often brittle nature, neat PIM HFs are rarely reported [55], and they are usually studied as TFCs [36,56] and/or as blends with other polymers [57,58]. Both approaches have the advantage that a low amount of PIM, or other expensive material, is necessary.

Another class of high free volume membrane materials with high chemical inertness, and thus high resistance to common organic solvents, are the glassy perfluoro-polymers. These polymers are often used in the form of thin-film composite HFMs due to their high cost and low mechanical resistance. Hyflon^®^ AD was supported on PAN and PEEK HF ultrafiltration membrane supports [59], and Teflon^®^ AF2400 was supported on commercial PP and sulfonated PES membranes [15]. Moreover, thanks to their hydrophobic nature they can be used as a coating to improve the water resistance of composite membranes as well [60].

### 2.3. Polymer Blends

**Figure 2:**
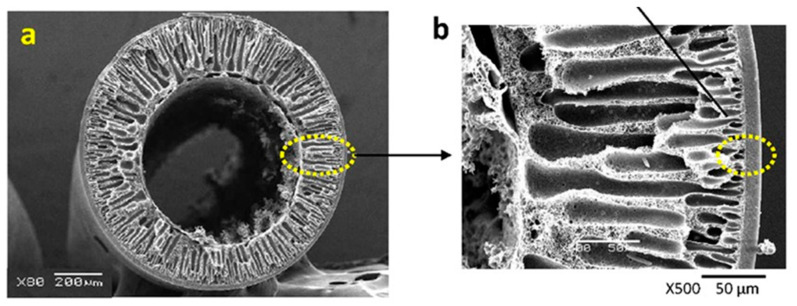
(**a**) Dual-layer HF of PBI/Matrimid^®^ polymer blend; (**b**) enlargement of Dual-layer HF of PBI/Matrimid^®^ polymer blend. Reprinted [62] with permission from Elsevier.

Polymer blends represent an appropriate method for producing HFMs applicable in gas separation because it can be economically advantageous, matching polymer with different properties in order to obtain advantages in terms of the trade-off between permeability and selectivity or mechanical and thermal resistance improvements. Different polymer blend materials have been studied for the preparation and characterization of gas separation HFMs, in both structure and configuration, as single-layer [61], dual-layer [1,62], or multi-layer HFs [63]. An example of a dual-layer HF and its enlargement are reported in Figure 2.

### 2.4. Hollow Fiber Mixed-Matrix Membranes (HF-MMMs)

HF-MMMs offer the opportunity to match and design innovative HFMs with the benefits of low cost and easily processable polymeric materials with the excellent transport performance of fillers such as zeolite [64], carbon nanotubes (CNTs) [65], metal–organic frameworks (MOF) [66,67], covalent organic frameworks (COF) [61], ionic liquids (ILs) and generic inorganic or organic fillers [47,68].

**Figure 3:**
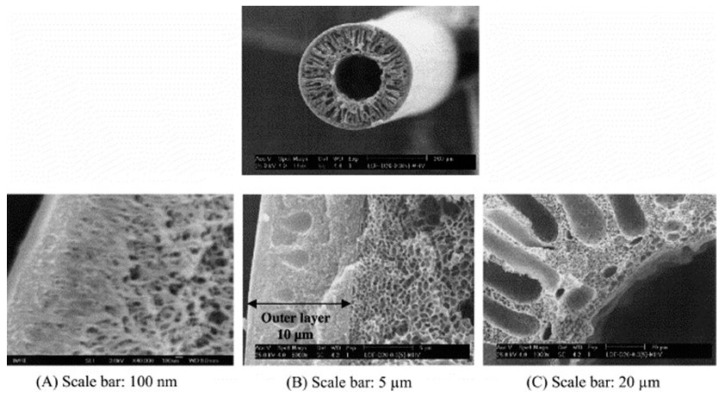
Cross-sections of 6FDA–durene–mPDA/PES dual-layer asymmetric hollow fibers [78] with permission from Elsevier.

A notable development was the introduction of ZIFs, a subclass of MOFs, as fillers in MMMs. Specifically, ZIF- 8 and ZIF-90-based MMMs were reported by Ordonez et al. [69] and Jones et al. [70]. The design of these new materials for gas separation had the objective of producing innovative membranes with enhanced permeability and selectivity, exceeding the Robeson upper bound limit. The addition of fillers into the dense selective layer can enhance in a different way the gas transport performance of the hollow fibers prepared just with the neat polymer. Firstly, the filler can have a strong effect on the diffusion promoting the permeability and, in some cases, even the diffusion selectivity when the diffusion is promoted only for the target gas [71,72]. On the other hand, the fillers can influence the solubility parameters, increasing the permeability due to the higher affinity for a specific gas. In this case, the selectivity will be more affected by the solubility selectivity [70,73]. For instance, considering the physical properties of the filler particles, spherical fillers promote better contact with the membrane matrix, enhancing the compatibility between the two materials. This improved interface contributes to better CO_2_ separation performance in mixed-matrix membranes (MMMs). On the other hand, lamellar fillers, with their elongated shape, create a tortuous pathway for gas molecules, further boosting the separation efficiency [74]. The uniform dispersion of fillers within the polymer matrix is crucial. Non-uniform dispersion can lead to the formation of defects that compromise the selectivity of the membrane. Therefore, the dispersion quality significantly impacts the overall CO_2_ separation performance of MMMs [75]. An example of cross-sections of HF-MMMs are reported in Figure 3.

In specific polymers like PIMs, which are prone to aging phenomena, the presence of fillers could influence the local polymer dynamics, mitigating the chain motion and the gradual loss of fractional free volume, which is characteristic for physical aging and loss in permeability, bringing long-term benefits on the gas transport properties [76].

The choice of the polymer and the filler particle loading are the most important parameters affecting the morphology and the performance of HF-MMMs. Issues in the fabrication of HF-MMMs can occur when the combination of fillers and polymer are inappropriate. This will cause undesirable morphologies at the interface between the polymer and fillers [77], commonly called “sieve-in-a-cage”, “pore blocking”, and “matrix rigidification”, leading to negative effects on the gas transport properties.

For this reason, the successful development of better-performing HF-MMMs needs a carefully matched polymer and filler combination to yield improved gas separation performance. Husain and Koros are pioneers in the fabrication of successful HF-MMMs for gas separation and they made different attempts to solve the issues related to the undesirable morphology of defected HF-MMMs [79]. In the fabrication of SSZ-13 zeolite/polyetherimide-based hollow fibers, they solved the problem of “pore blocking” due to the adsorption of solvent/nonsolvent in the zeolite cage by modifying the zeolite surface with Grignard reagent, which makes the zeolite hydrophobic and impermeable to coagulant water [79]. Moreover, chemical modification of the fillers’ surface is used to increase the compatibility between polymer phase and fillers [80]. Buddin et al. report the synthesis of 2D leaf-like ZIF-L and its modification using the ionic liquid [BMIM][BF_4_]. The nanosheet was then embedded up to 5 wt.% into the PES to form HF-MMMs to separate CO_2_/N_2_ gases [81]. This strategy can improve the compatibility at the interface, avoiding the formation of the “sieve-in-a-cage” morphology. With this aim, different types of fillers were fabricated, providing the incorporation of an organic or organometallic component such as ZIFs, MOFs, COFs, etc., in order to enhance the affinity for the polymer phase [82].

Roslan et al. [83] developed hollow fibers coated with multiple layers by integrating GO nanosheets into the selective coating layer composed of polyether block amide, i.e., Pebax^®^. The most efficient membrane exhibited not only reduced plasticization but also maintained a consistently stable performance throughout a 50 h operation. It shows impressive selectivities for gas pairs, specifically 53 for CO_2_/CH_4_ with CO_2_ permeance at 28.08 GPU and 8.05 for O_2_/N_2_ with O_2_ permeance at 5.32 GPU.

The advancement of high-performance hollow fiber mixed-matrix membranes (HF-MMMs) for gas separation hinges on the strategic combination of polymers and modified fillers, employing innovative surface chemistries and multi-layered designs to overcome morphological defects and achieve superior, stable separation efficiency.

## 3. Preparation Methods and Impact on HFMs Structures

### 3.1. Dry–Wet Spinning

**Figure 4:**
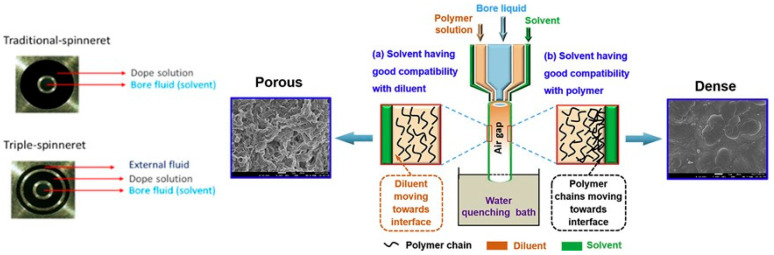
Traditional and triple-orifice spinneret with schematic representation of different HF’s surface structures obtained according to the different affinity of solvents for diluent or polymer chains when solvents are extruded at the contact interface using a triple-orifice spinneret. Adapted from [86] with permission from Elsevier.

Asymmetric hollow fiber (HF) membranes are fabricated using the “dry–wet” solution processing technique. This technique involves extruding a polymeric solution through a spinneret into an air gap (the “dry” stage) and then into a non-solvent bath (the “wet” stage), as shown in Figure 4. During this process, the polymer solution undergoes phase separation due to the exchange of solvent and non-solvent, resulting in the formation of the HF membrane. Traditionally, spinnerets with two holes offer limited control over the HF formation in the air gap. In 1994, Li et al. [84] introduced a new type of spinneret with three openings. This triple-orifice spinneret allows for the co-extrusion of the polymer solution, the non-solvent, and an external fluid. The outer fluid, inner dope, and bore fluid are fed into the spinneret separately by three syringe pumps. Sometimes, the outer and inner dope are pre-contacted before exiting the spinneret to enhance their integration. The advantage of using a triple-orifice spinneret is that it enables the preparation of dual-layer HF membranes in a single step. The deposition of the dense skin layer on top of the porous support can be controlled by adjusting the spinning parameters, offering flexibility in the membrane’s properties. Tepper et al. [85] introduce a scalable hollow fiber membrane fabrication methodology overcoming tedious assembly challenges. It comprises the simultaneous fabrication and integration of static mixers inside hollow fiber membranes by a single-step spinning process.

### 3.2. Melt Spinning

**Figure 5:**
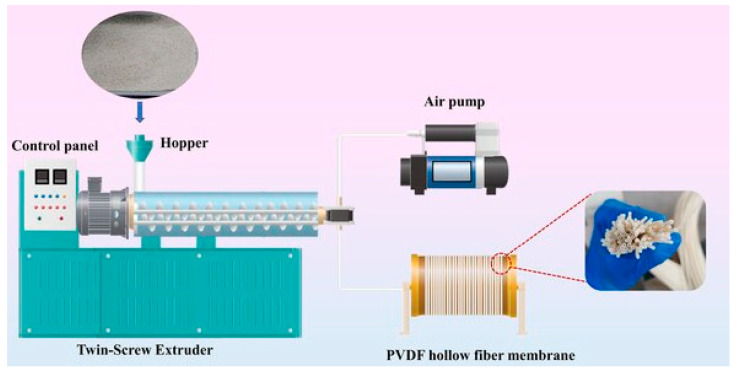
Melt spinning process for hollow fiber membrane preparation. Reference [91] with permission from John Wiley and Sons.

Melt spinning is the primary method for producing synthetic textile fibers like polyester and polyamide due to its ability to achieve high speeds and create small fiber dimensions through high draw ratios. However, it presents challenges for HFs, which require a cylindrical instead of spherical opening in the spinneret, with an inner tube for the bore gas. Figure 5 shows an example of the melt spinning hollow fiber membrane preparation process. Liu et al. [87] demonstrated melt spinning of dense PVDF HFs using a tube-in-orifice spinneret with inner and outer diameters of 3.6 and 5.2 mm, respectively, and air as the bore gas. Despite a high drawing rate of 70 m min^−1^, they only achieved membranes with a thickness of 115 µm and an inner diameter of 900 µm. Thinner membranes were obtained using a spinneret with an outer diameter of 0.21–0.25 µm [88], but this thickness still exceeds that of the dense skin achievable through the nonsolvent-induced phase separation (NIPS) process, around 0.2 µm [89]. Due to the low permeability of PVDF, melt-spun HFs are unlikely to be suitable for separation processes. Thinner poly(4-methyl-1-pentene) fibers, with outer diameters ranging from 40 to 90 µm and wall thicknesses of 8–20 µm, were obtained from a smaller spinneret using very high take-up speeds [90]. Under specific conditions, these fibers maintained relatively high selectivity (O_2_/N_2_ ≈ 4 and CO_2_/N_2_ ≈ 12). However, their thickness cannot compete with the extremly thin films achievable via NIPS, which remains the preferred method for dense HF membrane preparation.

### 3.3. Dip Coating and Dynamic Coating

**Figure 6:**
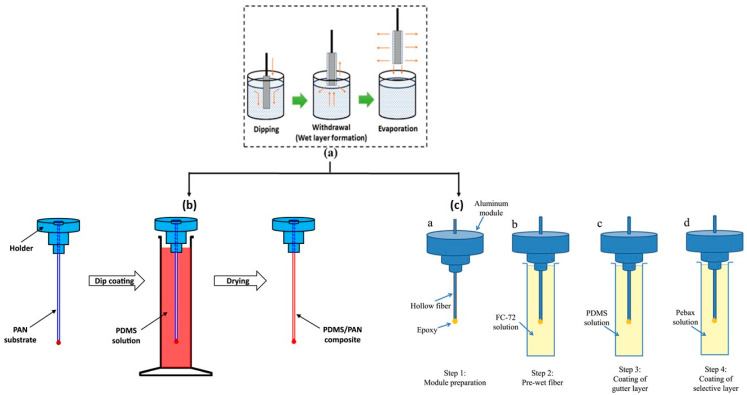
Scheme (**a**) of the dip coating process and schematic diagram of (**b**) the single dip coating membrane process [36] and (**c**) multi-layer dip coating membrane process [48], with permission from Elsevier.

Two commonly employed techniques for applying a thin, dense selective layer onto a porous support are dip coating and crossflow filtration deposition. These methods offer flexibility in adjusting the film thickness and the resulting permeability by manipulating parameters such as polymer solution concentration, viscosity, contact time, and the number of coating treatments. Dip coating involves immersing the porous support into the polymer solution, gradually lifting it at a controlled speed, and allowing solvent evaporation, as shown in Figure 6.

**Figure 7:**
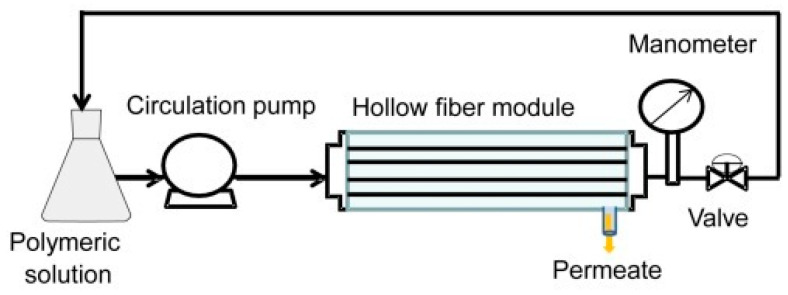
Illustrative representation of the dynamic coating method [49].

The packing density of hollow fibres and their self-supporting make them suitable for industrial applications. Hollow fiber membranes prepared using the spinning technique often may have pores or “pinholes” on their surface, which reduce selectivity. Dip coating deposits a dense layer that seals these defects. By coating the porous support with a material that has a higher affinity or a different diffusion rate for the target gases (e.g., CO_2_ compared to N_2_ or CH_4_), selectivity can be significantly increased. Creating an extremely thin selective layer reduces mass transfer resistance, thereby increasing gas permeability. The membranes produced have a thin selective layer supported by a thicker porous structure that provides mechanical strength. Achieving a uniformly thin, defect-free selective layer is crucial but can be complex. Parameters such as the coating solution concentration, immersion time and speed, and curing temperature must be carefully optimized [92]. A specially designed continuous coating system (Figure 7) offers several benefits over traditional methods like direct immersion. It minimizes the need for manual intervention and produces a more even and consistent coating on the membrane. However, a potential drawback is that the membrane’s outer functional layer can be prone to sticking and rubbing during the coating process. This friction might damage the coating, causing it to break or peel off the substrate. Consequently, an additional layer of silicone rubber is often needed after coating, which can unfortunately decrease the membrane’s efficiency for gas permeation [93].

### 3.4. 3D Printing

Three-dimensional printing technology is changing the gas separation membranes production. This innovative approach allows us to build membranes with high precision and intricate designs, a significant leap beyond conventional manufacturing methods. By constructing membranes layer by layer, 3D printing offers fine control over their internal structures, leading to a notable enhancement in their ability to separate gases [94,95]. Using these techniques, inks or bioinks are either applied after manufacturing or directly squeezed out of an extrusion nozzle to create hollow fibers using two main 3D printing methods. One involves a rotating vertical extrusion nozzle and a vertically moving horizontal substrate. The other uses a horizontally rotating rod with an extrusion nozzle moving along its axis. While 3D printing allows for precise hollow fiber fabrication, the process is slow, costly, and constrained by resolution and material limitations [96]. In the literature there are more studies about 3D-printed adsorbents for CO_2_ capture, like zeolites [97], amines [98], MOFs [99], carbon-based adsorbents [100]. Additive fabrication, by allowing for quick prototyping and scalable production, effectively tackles the limitations of conventional fabrication techniques. This aligns perfectly with the aim of creating membranes that are innovative, efficient, and sustainable. The combination of 3D printing and nanotechnology opens up exciting new possibilities for overcoming long-standing hurdles in membrane fabrication. This includes achieving a more uniform distribution of filler materials and significantly reducing defects, leading to higher-performing membranes [101].

### 3.5. Dual-Layer Asymmetric Hollow Fiber Membranes

**Figure 8:**
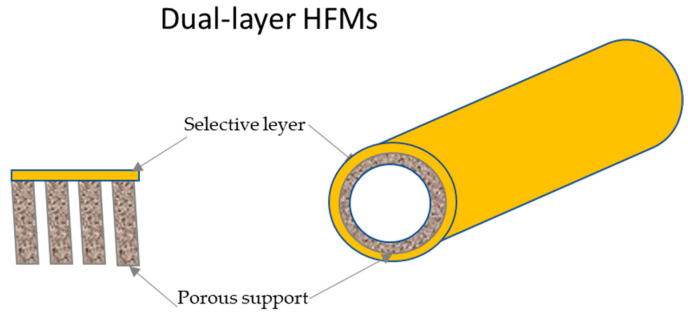
Dual-layer composite HF structure.

The structure and morphology significantly influence the gas transport properties and effectiveness of hollow fiber membranes. The membranes used in gas separation are fundamentally of the dual- or multi-layer type, as they always consist of a porous support and a thin, selective dense layer. They are never single-layer hollow fibers, also known as symmetric HFs, but only have an asymmetric hollow fiber structure. Two main categories of asymmetric hollow fiber membranes exist: Loeb–Sourirajan membranes, where the porous support layer and relatively dense surface layer form simultaneously from the same material, and composite membranes. Composite membranes consist of a microporous support layer coated with one (dual-layer HF) or more thin layers of a different polymer (multi-layer HF) for separation (Figure 8).

The use of a triple-orifice spinneret allows for the production of fibers with distinct layers composed of different polymers, enabling adjustments to the final morphology of the HF membranes. For instance, Chung’s group employed this technique to create Matrimid^®^/PBI polymer blend dual-layer HF membranes, resulting in a structure with a thin dense-selective outer layer and an inner supporting layer characterized by open cell pores and finger-like voids [62]. The morphological variations between the layers are attributed to differences in the chemistry of the dope components and the coagulation agent. Additionally, Fang et al. [102] investigated the impact of mixed diluents during HF preparation using a triple-orifice spinneret. By controlling the affinity of the solvent for diluents and polymers, they were able to tailor the network morphology of the fibers. Depending on the solvent compatibility, co-extrusion resulted in either increased membrane surface pore size and porosity or decreased pore size, leading to the formation of a dense layer suitable for gas separation [102].

Using a novel approach in the thermally induced phase separation (TIPS) process, PVDF hollow fiber membranes with distinctive spherulite surface structures were created. This new method involved a triple-orifice spinneret and solvent co-extrusion in the outermost channel, allowing us to tailor the membrane surface from a dense to a highly porous structure with large pores [102]. Zhang et al. [103] developed a one-step surface entrapment of an amphiphilic copolymer onto a PVDF_HFMs surface prepared via a thermally induced phase separation (TIPS) method. By extruding a poly(styrene)-b-poly (ethylene glycol) methacrylate (PS-PEGMA) solution at the outermost layer of a triple-orifice spinneret, entrapment of the amphiphilic copolymer onto the PVDF HFM was performed [103].

Similarly, an innovative approach in hollow fiber membrane engineering has also been explored for gas separation, utilizing a triple-orifice spinneret to optimize structural properties and regulate gas transport. A triple orifice spinneret was employed to spin polyimide hollow fibers specifically for their use in separating gases. The membrane’s structural properties were optimized to create a porous external region and a thin internal skin layer, thereby regulating gas transport. Data from gas permeation rate measurements and morphological characterization were integrated to assess membrane performance [104]. Of the various triple-orifice spinneret designs, one particularly interesting method is the co-extrusion technique that has been found to be an effective method to regulate the membrane pore size. Surface structure co-extrusion technology was employed to modify the outer surface structure of the polyketone (PK) hollow fiber membrane to enhance membrane permeance. By extruding various solvents at the outermost layer of the triple-orifice spinneret, besides the interaction among PK, diluent, and extruded solvent, it was emphasized that the viscosity of the outer solvent significantly influenced the penetration process and consequently affected the microstructure of the membrane surface [105].

Karousos et al. [106] employed a new dip coating method called “drop-casting under flow” to fabricate dual-layer HF-MMMs for the separation of CO_2_ from CH_4_ and CO gases. The substrate consisted of BTDA-TDI/MDI (P84) co-polyimide-based hollow fibers, prepared by dry–jet wet spinning, while the selective layer was composed of 5% of poly(ether-block-amide) (Pebax^®^1657). The effect of pressure on separation performance was systematically investigated, with transmembrane pressures of up to 10 bar being applied at a temperature of 298 K. The membranes exhibited excellent performance in separating CO_2_ from both CH_4_ and CO, achieving selectivities of up to 110 for CO_2_/CH_4_ and 48 for CO_2_/CO.

### 3.6. TFC-Hollow Fiber Membranes

The dry–wet spinning method allows for the production of membranes with a completely dense skin. This skin typically measures between a few hundred nanometers and several microns thick on the surface. TFC-HFs are distinguished by a thin selective layer, typically ranging from 0.1 µm to 1 µm thick, reinforced by a porous substrate. Using ultra-thin composite HF membranes ensures high gas fluxes, thus ensuring the economic feasibility of membrane gas separation processes. In contrast to integral asymmetric membranes prepared using the Loeb–Sourirajan technique, composite membranes offer distinct advantages, including fewer constraints on material mechanical properties, increased process adaptability, and reduced material consumption for the selective layer (0.1–2 g m^−2^). This facilitates the development of high-performance HFs for gas separation, even utilizing unconventional or expensive materials [107].

Zhao et al. [108] have prepared a TFC-HFMs for CO_2_/N_2_ separation by selectively coating the inner surface (lumen) of PES hollow fibers with polydimethylsiloxane (PDMS). This is followed by pressurizing the fibers. The optimal PES-PDMS TFC membrane can achieve a CO_2_ permeance of 2150 GPU and a CO_2_/N_2_ selectivity of 20 when the operating pressure was between 20 and 22 bar, which holds good potential for post-combustion CO_2_ capture. The high separation performance arises from a combination of the stretched PES and PDMS layers [108].

Recent studies indicate that incorporating organic and inorganic materials into the coating layer can improve its performance. Jia et al. [109] propose a second repair growth method for ultra-thin ZIF-8 membranes on a flexible polymer hollow fiber support (ZIF-8@PAN HF membranes). The optimized performance of ZIF-8@PAN HF membranes reaches H_2_ permeance of 662 GPU and H_2_/N_2_ selectivity of 26.7. Furthermore, an environmental impact analysis, specifically a life cycle assessment, of ZIF-8 membranes designed to select hydrogen, indicated that the second repair growth technique results in the least environmental harm when compared to three other methods usually discussed in the literature largely because it works at room temperature; this characteristic enhances its energy efficiency and substantially diminishes its carbon footprint. Consequently, this method exhibits the minimal global warming potential (expressed as kg CO_2_ equivalents) in comparison to the other three methods, which necessitate greater energy input.

Examples of TFC-HFs fabricated through the dry–wet spinning process are reported in Table 1.

#### 3.6.1. Multi-Layer TFC-HF

**Figure 9:**
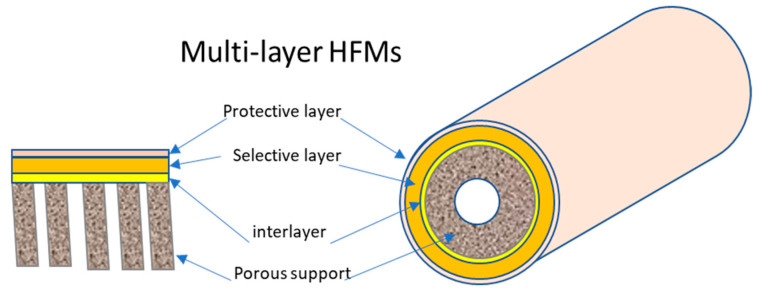
Multi-layer thin-film composite HF structure.

Multi-layer thin-film composites are constructed by layering different materials, including a porous support, an intermediate layer, a selective film, and occasionally a protective layer (Figure 9). The porous support, typically composed of cost-effective materials like PI, PSf, or PAN, provides mechanical strength without hindering mass transport across the membrane. Therefore, optimizing the substrate’s properties and morphology in composite membranes is as crucial as forming a defect-free selective layer. The substrate must possess a highly porous bulk structure with a smooth surface, small pores, high surface porosity, and a narrow pore size distribution. The intermediate layer, also known as the gutter layer, prevents the dilute solution of the selective polymer from penetrating the porous structure and blocking the pores, while enhancing adhesion between different materials. Materials such as PDMS and PTMSP, known for their high permeability, are commonly used for the intermediate layer, with PDMS often preferred due to its resistance to physical aging. The selective layer, where separation occurs, defines membrane efficiency and can be located on the inner surface or outside of the fibers. Coating the selective layer externally may require a protective layer to safeguard its delicate nature and heal any defects, thus improving membrane selectivity. TFC-HFs are categorized as dual-layer or multi-layer, with examples including ultra-thin PDMS/PAN composite HF membranes with high CO_2_ permeance and selectivity developed by Liang et al. [36], and multi-layer TFC-HFs containing a defect-free PIM layer reported by Chung and Xu [61].

Multi-layer coated PSF hollow fibers were fabricated by incorporating a graphene oxide (GO) nanosheet into the selective coating layer made of Pebax^®^ with a gutter layer of PDMS between the substrate and the Pebax^®^ layer to prevent the penetration of the Pebax^®^ coating solution into the membrane substrate. The optimal performance for the multi-layer coated membrane was observed with the inclusion of 0.8 wt.% GO in the Pebax^®^ layer. This composition yielded superior gas separation, with CO_2_/CH_4_ selectivity improving by 56.1% and O_2_/N_2_ selectivity by 20.9% over the GO-free membrane [83].

Choi et al. [116] created multi-layered thin-film composite hollow fiber membranes for helium extraction. A selective polyamide layer was formed on the surface of polyacrylonitrile hollow fibers using a technique called interfacial polymerization. This involved reacting 1,3,5-benzenetricarbonyl trichloride with m-phenylenediamine. To seal any defects in the polyamide layer, the membranes were dipped in a solution of poly[1-(trimethylsilyl)-1-propyne] (PTMSP). The resulting membranes exhibited high selectivity for helium over carbon dioxide, with values ranging from 30 to 38. The separation factor varied between 2.3 and 11.9, and helium permeance ranged from 3.4 to 46.2 gas permeation units, depending on the operating conditions in a mixed gas system [116].

Liang et al. [36] created a three-layer membrane using a dip coating method. This membrane, composed of PIM-CD, PDMS, and PAN, was free of defects. It exhibited impressive gas separation performance, with oxygen and carbon dioxide permeances of 69 and 483 (GPU), respectively. The selectivity for O_2_/N_2_ and CO_2_/N_2_ was 3.2 and 22.5, respectively. When tested with air and flue gas, these selectivities improved to 4.2 and 29.5, respectively. The PDMS gutter layer played a vital role in the preparation of the membrane by reducing the negative effects of solvents, improving the adhesion of the PIM layer to the support, and optimizing gas transport through the membrane [36]. Zakariya et al. [117] created composite membranes by layering NH_2_-MIL-125(Ti)/Pebax^®^ onto a base of PDMS/PSf. Subsequently, they evaluated the gas separation performance of these composite structures, with a particular emphasis on separating high-CO_2_ content gas streams under varying pressure and CO_2_ concentration levels [117]. These advancements demonstrate the potential for enhancing gas separation performance using innovative membrane designs and materials.

These diverse approaches, employing multi-layered designs, interfacial polymerization, and novel material combinations like PIM-CD/PDMS/PAN and MOF/Pebax^®^ composites, collectively highlight the significant progress and promising future of engineered membranes for advanced gas separation applications, especially for challenging mixtures like helium and high-CO_2_ streams.

#### 3.6.2. TFCs by Dip Coating and Dynamic Coating

González-Revuelta et al. [19] compare two materials, PDMS and Matrimid^®^, to study three different hollow fiber membranes for O_2_ separation from air. In the first step, hollow fiber membranes were prepared using a Matrimid^®^ polymer by a spinning process. In the second step, by a dip coating method, a thin layer of a PDMS, was applied to a hollow fiber support made of PVDF. The final phase of the study involved the performance assessment of a dual-layer hollow fiber membrane fabricated from Matrimid^®^ and PDMS [19].

Glassy polyacrylonitrile (PAN) is another interesting polymer used to produce hollow fiber membranes. Liang et al. proposed a novel post-crosslinking method to create a hollow fiber composite membrane with a high-viscosity, crosslinked PDMS matrix. The influence of various crosslinking conditions and spinning parameters on the membrane’s inherent viscosity and substrate morphology was studied. The resulting defect-free membrane exhibited exceptional O_2_ and CO_2_ permeances, exceeding 1000 and 5000 GPU, respectively, along with impressive O_2_/N_2_ and CO_2_/N_2_ selectivities of approximately 2 and 11, respectively [118].

Examples of TFC-HFs produced via dip coating are provided in Table 2.

In the dynamic coating method, also called the crossflow filtration deposition process, the polymer solution can be introduced into the inner part of the porous support fibers using a circulating pump. In membranes prepared by this technique, the outer porous support acts as a protection layer for the selective coating. Another advantage of crossflow coating is its capability to coat multiple fibers simultaneously, especially when they are already installed in a membrane module. Esposito et al. [49] demonstrated the efficacy of this dynamic coating technique in fabricating thin-composite Pebax^®^1657/PAN HFs for CO_2_/CH_4_ separation. They explored the impact of various experimental variables on both the thickness of the selective layer and the gas transport properties of the resultant HFs. They achieved TFC-HF membranes with a minimum thickness of 0.5 µm, a CO_2_/CH_4_ selectivity of roughly 6, and a CO_2_ permeance of 48 GPU. Lasseuguette et al. [15] uncovered the relationship between the thickness of the liquid film coated onto a fiber and factors such as coating velocity, the physical and chemical characteristics of the liquid, and coating geometry. They identified several operational regimes, including two unstable ones where a thin and uniform polymer layer could not be achieved, and two stable ones, as follows: the viscocapillary regime, applicable in laboratory settings, and the high-velocity boundary layer regime, preferred in industrial contexts [15]. These studies collectively demonstrate the versatility of dip coating and dynamic coating techniques for creating high-performance composite hollow fiber membranes using diverse polymer combinations (PDMS, Matrimid^®^, PAN, and Pebax^®^) to achieve efficient gas separations, with a focus on optimizing layer thickness and material properties for enhanced selectivity and permeance.

### 3.7. Green and Sustainable Development of HFMs Preparation

The NIPS technique is the most common and traditional method to fabricate the porous support for the gas separation HFMs that utilizes traditional organic solvents. For this reason, there is a pressing need for further enhancing and refining this membrane technology to meet the demands of contemporary environmental protection, resource efficiency, and specialized separation requirements in challenging conditions. In response to environmental concerns, significant efforts have been made in researching environmentally friendly solvents as alternatives to the conventional toxic ones for the HFMs manufacturing processes. The choice of the most suitable replacement for traditional solvents in HF production is essential to prioritize factors such as their favorable toxicological profile and specific physical properties. These include complete solubility in water, high boiling point, and low molecular weight. The latest innovative solvents identified for creating hollow fiber membranes with their gas transport properties are reported in Table 3.

TEP is emerging as a promising candidate for fabricating hollow fiber membranes due to its low toxicity and compatibility with PVDF. Abed et al. [122] obtained HFMs with interconnected structure using TEP as the solvent via a single-step phase inversion method. The hollow fiber membranes produced from the PVDF/TEP solution exhibited impressive mechanical characteristics with a morphology that does not allow water flux. However, it could still be sufficiently permeable to gases and used as a smooth surface for the deposition of a selective thin layer of another polymer for TFC-HFMs fabrication [122]. In this field, Theodorakopoulos et al. [123] conducted innovative research, suggesting GBL as a safe and non-toxic solvent for the fabrication of Polyimide HFMs for CO_2_/CH_4_ separation [123]. The achieved performance was comparable to that of HFs prepared using highly toxic solvents, with a CO_2_/CH_4_ separation factor of about 26. HFMs based on a thin selective layer of meta-polybenzimidazole (m-PBI) were created using ethanol as a sustainable solvent to dissolve the polymer by Sanchez-Lainez and his group [124]. This approach offers an eco-friendly alternative to the toxic and corrosive N,N-dimethylacetamide typically used, while enabling membrane processing at ambient temperature demonstrating great H_2_/CO_2_ separation performance under continuous operation conditions about 22 days at high temperature 180 °C [124]. Esposito et al. successfully prepared composite Pebax^®^/PAN hollow fiber membranes by using a green EtOH/water solvent mixture for the solubilization of Pebax^®^1657 and subsequent application as a coating layer using the dip coating techniques as discussed in the previous section [49]. Regarding the use of biopolymers for the fabrication of HFMs, finding examples of dual-layer asymmetric HFMs prepared through a single step is indeed rare, and there are few instances. It is easier to find studies where biopolymers have been used to prepare porous hollow fibers or directly for coating on a porous support to form the selective dense layer. Mubashir et al. investigated CA hollow fiber membrane preparation conditions and found that under optimal spinning conditions, the membranes showed a selectivity of 7.9 for CO_2_/CH_4_ and 6.1 for CO_2_/N_2_ gas pairs, respectively, and a sponge-like structure [125]. Expanding on the successful use of Pebax^®^/PAN composite membranes for biogas treatment, recent advancements further highlight the potential of eco-friendly materials in hollow fiber membrane technology, with studies demonstrating effective gas separation performance using biopolymers like cellulose acetate and cellulose triacetate/diacetate in both single- and dual-layer configurations.

Raza et al. [34] have created a new type of membrane made from a mix of two materials as follows: cellulose triacetate (CTA) and cellulose diacetate (CDA). This membrane is shaped like a hollow fiber and is designed to separate CO_2_ from other gases. They made both single-layer and dual-layer versions of this fiber using different spinning techniques. The best-performing dual-layer membrane, spun at a specific flow rate, allows CO_2_ to pass through at a rate of 45 (GPU) while effectively separating it from CH_4_ with a selectivity of 30.3. Compared to a single-layer membrane, this dual-layer version is 100% more efficient at letting CO_2_ through without compromising its gas separation efficiency.

Sunder et al. [126] prepared hollow fiber mixed-matrix membranes (HF-MMMs) by combining CTA polymer with amine-functionalized metal–organic framework (NH_2_-MIL-125(Ti)) filler. This combination was specifically designed to enhance the separation of CO_2_ and CH_4_ gases. Both CTA and NH_2_-MIL-125(Ti) are recognized for their strong affinity for CO_2_ molecules [125,126]. This builds on efforts to enhance gas separation, while Hollow Fiber Mixed-Matrix Membranes (HF-MMMs) leverage the affinity of polymers and MOFs for CO_2_ to separate CO_2_/CH_4_, novel carbon hollow fiber membranes (CHFMs) derived from cellulose precursors achieve even higher CO_2_ permeability and selectivity through their unique microporous structure.

Novel carbon hollow fiber membranes (CHFMs) are prepared based on the cellulose hollow fiber precursors spun from cellulose–EmimAc/DMSO solutions. The CHFMs exhibited a micropore volume of 0.15 cm^3^ g^−1^ and an average pore size of 5.9 Å. This structure yielded a high CO_2_ permeability of 239 Barrer and a CO_2_/CH_4_ selectivity of 186. When tested with a 10% CO_2_-90% CH_4_ mixture at 28 bar, the CHFMs demonstrated an impressive selectivity of 75 [127].

Finding the application of biopolymers in conjunction with eco-friendly solvents for crafting hollow fibers poses an even greater challenge and only a few biopolymers have been used in combination with less toxic or non-toxic solvents. To obtain a bio-based poly(lactic acid) (PLA) hollow fiber membrane, Moriya et al. [128] employed DMSO at a high temperature of 90 °C to maintain the PLA solution at a low viscosity (~20% by weight). This DMSO serves as a safer alternative due to its high boiling point (189 °C at 760 mmHg) and very low vapor pressure [128]. Despite the potential benefits of these alternatives, replacing traditional solvents or using biopolymers or combination of both, without compromising the optimized membrane preparation protocol, remains a significant challenge.

### 3.8. Critical Aspects in the Preparation of HFMs for Gas Separation

**Figure 10:**
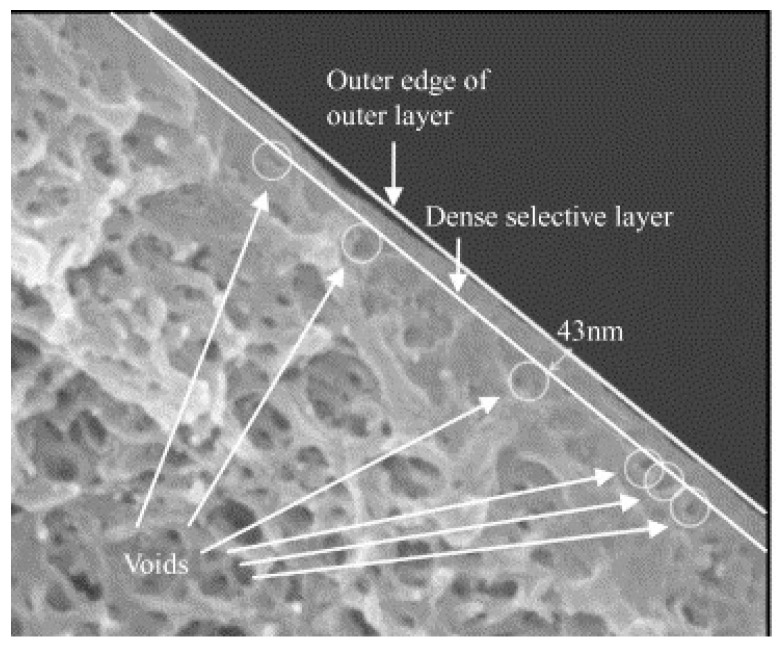
Ultra-thin selective layer [121], with permission from Elsevier.

The goal during HFM preparation is to achieve very thin, defect-free dense layers, as membrane integrity determines selectivity. The thickness of the dense layer inversely affects membrane productivity. Careful control of the parameters affecting morphology during preparation is essential to achieve the desired transport properties. The critical aspects of TFC-HF fabrication include preventing selective polymer penetration into the porous support and addressing or avoiding defects in the selective layer. The former minimizes mass transport resistance, while the latter ensures optimal gas transport properties and selectivity. Therefore, optimizing the morphology of the porous support is as crucial as forming the thin selective layer. Several methods have been developed to minimize the intrusion of the selective polymer into the porous support. In the pre-wetting technique, the porous support is pre-soaked with a non-solvent or a solvent that does not mix with the polymer solution before coating. For instance, Li et al. [129] achieved promising results by coating PAN HFs with PDMS, resulting in a CO_2_ permeance of 3700 GPU and CO_2_/N_2_ selectivity of 10. They prevented polymer intrusion by partially crosslinking PDMS and pre-wetting the PAN support with Fluorinert 72 or deionized water before dip coating. Vacuum-assisted dip coating, as demonstrated by Bazzarelli et al., yielded thicker SBS layers compared to standard dip coating procedures [130]. Li et al. [129] achieved ultrathin HFs with a thickness of 0.04 µm (Figure 10) by coating unselective PES HFs with a highly permeable silicone layer, resulting in an O_2_/N_2_ selectivity of 6.0 and a O_2_ permeance of 10.8 GPU. Additionally, the silicone layer can serve as a protective coating against sticking or mechanical damage, especially for selectively coated fibers. Achieving defect-free selective layers necessitates the meticulous examination of factors such as polymer concentration, its compatibility with the support material, and the rate of solvent evaporation. Moreover, ensuring a smooth surface on the porous support is imperative to ensure a homogenous distribution of the polymer solution during coating. A strategy to repair defects is to coat the selective layer with a highly permeable silicone layer [121].

## 4. HF Properties and Characterization

### 4.1. Mechanical Properties

The successful industrial implementation of HF membranes of adequate mechanical strength is crucial, as operating pressures can vary significantly depending on the specific gas separation process. For instance, the pressure can range from 10 to 15 bar for biogas separation to over 50 bar for applications like O_2_/N_2_ separation [131]. Moreover, the rigidity of the polymer significantly impacts on transport resistance and plays a crucial role in gas separation performance as observed by Hirayama et al. [132]. A strong correlation was found between the diffusion coefficient and the storage modulus in polyimide-based membranes, where the storage modulus represents an indicator of segmental mobility. Increased polymer rigidity results in lower diffusion coefficients, which is also associated with the Cohesive Energy Density [132]. This trend was also observed for light gases in PIMs, where high rigidity contributed to increased size selectivity [133]. Additionally, aging processes lead to a gradual decrease in the free volume in the polymer matrix, increasing chain packing density and reducing molecular mobility. This phenomenon contributes to polymer stiffening caused by a molecular rearrangement [134,135], as reported by Chung and Fuoco for fibers of polyethersulfone. Furthermore, the fibers spun with high shear rates show a higher increment in tensile strength compared to those spun with low shear rates [134]. One way to accelerate the aging and enhance the thermodynamic stability of membranes is using heat treatments. In some polymers, sintering temperatures facilitate the formation of smaller molecular sieving pores, affecting tensile strength, Young’s modulus, and elongation at break [136]. In MMMs, the presence of fillers such as nanoparticles can decrease elongation at break and increase rigidity, sometimes making the material more brittle [137]. However, as demonstrated by Modi et al., [137] the incorporation of functionalized CNTs in HFMs improved both the thermal stability and mechanical strength. This can be attributed to the enhanced interfacial compatibility between oxygen-containing functional groups on CNTs and sulfone groups of the PES matrix [138]. In general, the good distribution of the filler and strong filler–polymer matrix interactions can be the main factors contributing to improved mechanical properties. Moreover, the active functional groups attached on the GO planar surface contributed to create interfacial interaction and strong bonding with the polymer matrix, thereby enhancing the improvement of tensile strength [139]. The development of hollow fibers with dual-layer mixed-matrix materials has gained significant attention in the field of gas separation due to their potential for enhancing both performance and mechanical properties. Notably, dual-layer PES–beta zeolite/PES–Al_2_O_3_ mixed-matrix hollow fiber membranes have shown significant improvements in both gas separation efficiency and mechanical strength [140].

An approach to improve the transport parameters and remodel the mechanical behavior is the use of the ILs, which, in general, reduce the rigidity of membranes by decreasing polymer intra-chain interactions and entanglements, leading to increased elongation [141,142]. In membrane fabrication, polymer selection and membrane morphology play a critical role, as they determine mechanical strength and overall performance. The arrangement of the molecules helps to improve the mechanical properties of the hollow fibers, boosting also their gas separation performance. Spin-line stresses play an important role in aligning the molecules, as reported in previous works [143,144]. Conversely, the presence of macrovoids weakens the membrane, making it susceptible to damage under compression [145]. Controlling void formation, however, enhances membrane stability, leading to improved performance under high-pressure conditions [146]. Microporous materials, such as thermally rearranged (TR) polymers, pose additional challenges related to mechanical stability, as they are often brittle and require careful handling. TR polymers show low elongation at break, which further decreases with increased thermal rearrangement temperature [147]. Nevertheless, spiro-TR-PBO-based polymers are among the most mechanically stable, showing high elongation even after thermal rearrangement, outperforming PIM-1, which lacks thermal treatment [148]. The reduced mechanical stability of PIM-1 is likely attributed to the rigid dioxane unit, which restricts polymer chain entanglement. This suggests that the incorporation of specific functional groups could enable the design of mechanically robust hollow fibers based on their microporous structure. Finally, the fabrication of ceramic membranes relies heavily on optimizing both the composition and processing conditions. In particular, key factors influencing the mechanical properties of HFMs include sintering temperature, particle size, and ceramic loading [149,150].

### 4.2. Swelling/Plasticization

A plasticizer (or softener) is a substance added to a material to make it more flexible, workable, or stretchable. A plasticizer may reduce the melt viscosity, lower the temperature of second-order transitions, or lower the elastic modulus of the product [74]. They can be broadly classified into two categories as follows: internal and external. Internal plasticization entails chemically modifying the polymer’s structure to improve its flexibility or low-temperature behavior. External plasticization, on the other hand, involves adding a distinct substance to the polymer matrix to achieve the same effect. External plasticizers are commonly employed to either adjust the physical properties of final products like PVC tubing or to facilitate the manufacturing process by reducing the significant energy consumption required for mixing highly viscous substances [151].

Compressed fluids like CO_2_, N_2_O, propane, and C_2_H_4_ can be dissolved in polymers to a considerable extent when exposed to elevated pressures [152]. When these fluids are absorbed, the polymer undergoes a transformation. The polymer matrix swells, leading to an increase in both free volume and chain mobility. Free volume refers to the empty spaces within the polymer matrix that allow for the movement of polymer chains [153]. The introduction of high-pressure fluids has been demonstrated to induce substantial decreases in the glass transition temperature (T_g_) across a wide spectrum of polymeric materials [154]. Gas-induced plasticization can render an initially rigid, glassy polymer more pliable, similar to a rubber or liquid. By reducing the T_g_ and viscosity, new processing avenues emerge for the softened polymer melts. These new processing windows can potentially lower energy costs and increase process efficiency [155].

The plasticization of an amorphous polymer by CO_2_ has three distinct regions. In region I, the swelling of the polymer matrix by the dissolved gas dramatically lowers T_g._ This drop in T_g_ can occur even with small concentrations of CO_2_ in the matrix (1–5 wt.%). In region II, a constant T_g_ is observed at significantly lower temperatures than that of the pure substance. The large hydrostatic pressures generated to increase the solubility of the CO_2_ dominates in region III. The loss of free volume observed by the compression of the matrix by hydrostatic pressure results in a T_g_ increase. The dimensions of each region, as well as the degree of plasticization within it, are influenced by the level of matrix swelling, the polymer’s compressibility, and the solubility of CO_2_ in the polymer.

The phenomenon of plasticization has been more extensively researched in homogeneous dense membranes than in microporous asymmetric membranes, with particular emphasis on their influence on CO_2_ selectivity.

The effect of CO_2_ conditioning on asymmetric hollow fiber aromatic PI membranes, consisting of a 0.05–0.2 μm-thin skin layer and a 50–200 μm porous sub-layer, was investigated. The membranes were subjected to a feed pressure of 8.6 bar for 48 h and subsequently analyzed for oxygen permeability. A significant 50% enhancement in O_2_ permeation was observed for the asymmetric membrane compared to a homogeneous dense film (13.5%), while preserving O_2_/N_2_ selectivity. The improved performance can be attributed to the distinct molecular structures of the thin skin layer on the asymmetric membrane compared to the homogeneous dense film when exposed to CO_2_ treatment. During conditioning, the process may partially untangle the interconnected polymer chains, leading to an increase in free volume within the system. The high solubility of CO_2_ in the matrix nodules causes swelling and reduces the effectiveness of the dense thin skin layer on the asymmetric membrane, resulting in significantly higher gas permeation [156].

To enhance the durability of membranes, it is essential to create polymers that resist plasticization. This involves designing polymers that have a low affinity for plasticizer molecules, limiting their absorption into the membrane structure. Additionally, reducing the solubility of feed components in separation systems is crucial to prevent plasticization. To mitigate the solubility of gas and vapor molecules, operating conditions such as feed temperature and mixture ratio can be adjusted.

An example can be represented by the introduction of fluorocarbons into the feed that can mitigate the plasticizers caused by the expansion of CO_2_. For example, asymmetric polyamide-imide (PAI) hollow fibers, characterized by robust intramolecular and intermolecular hydrogen bonds, exhibit superior resistance to plasticization when exposed to hydrocarbons such as ethylene (C_2_H_4_) and fluorocarbons like tetrafluoroethylene (C_2_F_4_) and difluoroethylene (C_2_H_2_F_2_) [157]. Some commercial membranes are known for their resistance to plasticization and are used in both gas and solvent separation processes. However, the number of such membranes on the market is still limited. Most commercial membranes need to be modified to improve their resistance to plasticization before they can be used in industrial settings.

The Torlon^®^ membrane, a commercially available asymmetric hollow fiber membrane made of polyamide-imide (PAI), demonstrated consistent performance in supercritical conditions. When exposed to a mixture of 90% CO_2_ and 10% CH_4_ at a pressure of 84 bar and a temperature of 35 °C, the membrane achieved a CO_2_/CH_4_ selectivity of 44 and O_2_/N_2_ selectivity of 7.7. The polymer chains in the membrane formed strong hydrogen bonds between nitrogen-hydrogen (N-H) groups and carbonyl groups (C=O). These bonds acted like molecular glue, holding the chains together and preventing them from moving apart under pressure. This increased the membrane’s resistance to plasticization, allowing it to withstand higher operating pressures. Torlon^®^ could handle pressures exceeding 76 bar, significantly higher than the 27 bar limit of Ultem^®^ and the 12 bar limit of Matrimid^®^. However, this increased rigidity also hindered the movement of CO_2_ molecules through the membrane, reducing its permeability to 0.47 Barrer compared to 1.3 Barrer for Ultem^®^ and 12.5 Barrer for Matrimid^®^ [110].

In comparison to commercial CA and Matrimid^®^ membranes, commercial poly(phenyleneoxide) (PPO)_HFs sourced from Parker Gas (Etten-Leur, The Netherlands) demonstrated a reduced susceptibility to plasticization when subjected to a pressure of 16 bar. However, when the PPO membrane was exposed to a gas mixture containing both CO_2_ and CH_4_, a significant competitive effect was observed. This competition, coupled with the presence of surface defects on the membrane, resulted in a decrease in mixed gas selectivity to a value below 10 [158].

Another aspect to consider that influences plasticization is chemical crosslinking. Chemical crosslinking occurs when two polymer chains connect through a covalent bond. This bond restricts the movement of the chains, creating a very stable network structure. The polymer chains can be linked together using special chemical groups that attract CO_2_ molecules. These groups, like ethers, alcohols, ketones, or acids, form hydrogen bonds with each other, holding the chains in place. This not only strengthens the membrane but also makes it more permeable to CO_2_. Additionally, bulky groups like those containing fluorine or multiple amine and alcohol groups can be added to the polymer chains. These bulky groups act as spacers, pushing the chains apart and reducing their movement. This increased distance between chains improves the membrane’s resistance to swelling (anti-plasticization) and allows for better CO_2_ transport. In essence, both the CO_2_-attracting groups and the bulky groups work together to create a more robust and efficient membrane for CO_2_ separation [159,160].

### 4.3. Thermal Properties

Some gas separation processes require operation across a wide spectrum of temperatures, ranging from high to low and even experiencing fluctuations. This necessitates the careful selection of materials that can consistently maintain their structural soundness and operational effectiveness within these defined operating boundaries.

As the operating temperature approaches the glass transition temperature of the polymeric material, an enhancement in polymer chain mobility ensues. In integrally skinned or composite membranes, this increased mobility can trigger a phenomenon known as compaction within the porous, asymmetric membrane structure. This compaction can adversely impact both selectivity and permeation rates. The underlying mechanisms for these performance losses can be attributed to alterations in the intrinsic properties of the polymer itself or to physical damage sustained by the dense, selective skin layer of the membrane [23].

A practical example where thermal conditions are a very important point is the recovery of helium from natural gas using membrane technology. This process encounters a significant obstacle in the form of membrane plasticization. This phenomenon leads to an increase in gas permeation rates but simultaneously diminishes the ability of the membrane to selectively separate helium from other gases. Thermal crosslinking has emerged as a promising strategy to mitigate the detrimental effects of plasticization. Wang et al. [161] have developed a novel dual thermally crosslinked asymmetric HFMs using a 4,4′-diamino-2,2′-biphenyldicarboxylic acid-containing copolyimide. Dual crosslinking via decarboxylation, achieved by heat treatment at varying temperatures, introduces C-C covalent bonds. This increases the interchain distance from 5.33 to 5.76 Å, resulting in hierarchical pore size distributions with ultra-micropores (5.6–6.8 Å) and micropores (7.0–9.5 Å). The formation of stable C-C bonds and the presence of bulky CF_3_ groups within the 2,2′-bis(trifluoromethyl)-4,4′-biphenyldiamine moiety impede substructure collapse by enhancing chain rigidity and rotational barriers. Gas transport properties in these crosslinked high free volume HFMs are effectively modulated by adjusting heat treatment temperatures. The PI-TFMB-HF@400 membrane exhibits a helium permeance of 25 GPU with a He/CH_4_ selectivity of 269. Moreover, these crosslinked HFMs demonstrate improved plasticization resistance. For instance, the PI-TFMB-HF@400 membrane shows only a 24% decrease in mixed gas CO_2_/CH_4_ selectivity and an 80% increase in mixed gas [He/(CO_2_ + CH_4_)] selectivity when exposed to a high-pressure (40 bar) ternary mixed gas feed of He/CO_2_/CH_4_ (0.3/49.7/50, *v*/*v*/*v*). Notably, the mixed gas [He/(CO_2_ + CH_4_)] selectivity increases with temperature [161].

High-performance HFMs derived from polybenzimidazole (PBI) were fabricated by the Jong Geun Seong groups with minimal defects and evaluated for their oxygen/nitrogen separation capabilities for the first time [162]. The microstructural and oxygen-selective gas separation properties of these PBI-based CMS HFMs were thoroughly investigated across a range of pyrolysis conditions. The PBI HFMs were pre-treated for 12 h at 250 °C to remove traces of residual solvent and/or water that may have been absorbed during the fiber spinning operation. Following pre-treatment, the fibers were heated to 400 °C at a ramp rate of 1 °C min^−1^, followed by an additional heating step at a reduced ramp rate (0.3 °C min^−1^) to the target temperature. All samples were held at their target temperature for 2 h prior to cooling. The final pyrolysis temperatures were varied from 580 to 850 °C in this study. The Precursor PBI HFM had an O_2_ permeance and O_2_/N_2_ perm-selectivity of 0.2 GPU and 1.0, respectively. After pyrolysis, both the O_2_ permeances and O_2_/N_2_ selectivities improved significantly at all pyrolysis temperatures except 850 °C. The best O_2_ permeance and O_2_/N_2_ perm-selectivity combination of 4.0 GPU and 8.5, respectively, was measured for the PBI-CMS HFMs fabricated at 650 °C [162].

### 4.4. Physical Aging

#### 4.4.1. Basic Principle

Physical aging is a process that affects all glassy polymer membranes, particularly those with high fractional free volume such as PIMs, and it is related to the global non-equilibrium state of the glassy polymer matrix that tends to relax over time [163], while no chemical changes occur. This relaxation process results in a very slow rearrangement of the polymer chains and affects various properties of the polymers, such as reduced local chain mobility, increased overall rigidity [134,135] or Young’s modulus [133,164], reduced fractional free volume and a general rearrangement in the free volume element distribution, which has a strong impact on the gas transport properties [164,165]. Permeation experiments on Matrimid^®^ asymmetric HF membranes with pure gases and with CO_2_/CH_4_ and CO_2_/N_2_ gas mixtures [134,165] showed faster aging for the thinner membranes. It was found that this is a general trend, and the gas transport properties change much faster in thin films [166], which makes it extremely relevant for thin-film composites or integrally skinned hollow fiber membranes with a thin selective layer. In asymmetric PES HF membranes, the aging is further complicated because it appears to depend on the spinning rate, and besides the permeability it affects numerous physical properties, such as the tensile strength and Young’s modulus [134]. Clarizia et al. observed a general reduction in permeability as a function of aging time for asymmetric PI HF membranes prepared by dry–wet spinning with triple orifice spinneret [167], and while the selectivity remained roughly constant for several gas pairs, the H_2_/N_2_ selectivity increased mainly due to a higher size selectivity.

Various mechanisms have been proposed as the basis for the aging process, such as the diffusion of free volume elements from the bulk polymer towards the surface [168]. This process is very slow, and it is especially relevant in very thin films [169]. McCaig and Paul suggested a combination of thickness-dependent diffusion of free volume [166]. The aging is not always related to the selective dense film alone; in asymmetric Torlon^®^ polyamide-imide membranes, a notable decay of the permeance was observed after exposure to high pressure He/CH_4_ mixtures and this was also ascribed to the partial compaction of the smallest pores (radius < 15 nm) of the porous support [50]. The gutter layer, which must necessarily consist of highly permeable polymers like PDMS or PTMSP in order to be effective, is also a critical factor. Thin films of PTMSP are very sensitive to aging, and although this can be mitigated by strategically choosing the type of filler materials for the glassy matrix that reduces aging [170], rubbery PDMS is often preferred for its stability.

#### 4.4.2. Mitigation of Physical Aging

From an industrial point of view, aging is an undesired phenomenon because of the general decrease in permeability, and its importance and complex nature was already recognized several decades ago [134]. The decreasing permeability compromises the stable process operation and therefore its mitigation is necessary, especially for high free volume polymers where the effect is stronger [76]. Polyethersulfone HF membranes are subject to substantial physical aging, and Yong et al. demonstrated that the aging process can be strongly reduced by the incorporation of trimethylphenyl units into the polymer chain [171].

Ma and Koros demonstrated that physical aging in ester-crosslinked polyimide hollow fiber membranes can be mitigated by periodic CO_2_-conditioning or by continuous permeation with CO_2_/CH_4_ mixtures [172]. The thermal crosslinking of PDMC-PI membranes for 2 h under vacuum at 200 °C is largely responsible for the partial suppression on the physical aging. Analogously, crosslinked thermally rearranged poly(benzoxazole-co-imide) hollow fiber membrane modules for post-combustion CO_2_ capture were found to exhibit reasonable long-term stability due to the crosslinking [172]. The TR and crosslinking process also stabilized the polymer against the negative effect of humidity, which caused a dramatic decrease in CO_2_ permeability for the hydroxyl-containing linear polymer [173].

Blending of PI with PES also resulted in increased aging resistance compared to neat PI, probably due to chemical interactions between the two polymers and/or due to the intrinsic strength of PES [173]. Analogously, PIM-1/Matrimid^®^ HF membranes were prepared at different blend ratios with the aim to create synergy between the high permeability of PIM-1 and the high selectivity and better aging resistance of Matrimid^®^. The PIM-1/Matrimid^®^ ratio of 10–90 yielded a good compromise between permeability and selectivity for O_2_/N_2_ separation (PO_2_ = 50 Barrer; O_2_/N_2_ = 6) [58].

Another approach to improve the membrane performance and mitigate the effects of physical aging in HF gas separation membranes is via stabilization with various filler materials. The presence of fillers in glassy polymers, prone to aging phenomena, could influence the local polymer dynamics, mitigating the chain motion and the gradual loss of fractional free volume of the surrounding matrix. In the case of intrinsically porous fillers, such as Metal–Organic Frameworks, the fillers themselves provide stable additional free volume available for transport. ZIF-8 and SIO_2_/ZIF-8 nanofillers embedded in polysulfone hollow fiber membranes successfully enhanced the CO_2_/CH_4_ and CO_2_/N_2_ gas separation performance [174]. Sutrisna et al. produced Pebax^®^/ZIF-8 thin-film composite HF mixed-matrix membranes with high operational stability due to the formation of hydrogen bonds between ZIF-8 organic ligands and polyamide chains, which significantly improved the linear glassy polymer chain stiffness, ensuring good operational stability under elevated pressures [175]. Jang et al. grew mesoporous silica onto polymeric hollow fiber gas separation membranes [176] and being thermally, chemically, and dimensionally highly stable, this might yield membranes that are much less prone to physical aging. Park and Jeong reported in situ growth of ZIF-8 on asymmetric 6FDA-DAM hollow fiber membranes with a promising propene/propane separation performance (i.e., separation factor of ~23.4 and propene permeance of ~2.15 GPU) with a stable separation performance of up to 25 days [177].

#### 4.4.3. Exploitation of Physical Aging

Physical aging can also have positive effects because the decrease in permeability is often accompanied by an increase in selectivity, and in those cases, controlled aging may be used to tailor the gas transport properties. This is not limited to polymer membranes but also to 6FDA:BPDA-DAM polyimide-based hollow fiber carbon molecular sieve (CMS) membranes [178], where so-called hyper-aging under the right conditions leads to a radical increase in selectivity of hydrogen over hydrocarbons, and better stability. Accelerated aging at higher temperatures may also stabilize the polymer with very strong initial aging, especially for PIMs that have previously been treated with MeOH to release the residual casting solvents and reset the sample history [133].

### 4.5. Modeling

Modeling plays a critical role in understanding and optimizing hollow fiber membranes. A variety of modeling approaches—ranging from empirical correlations to advanced molecular simulations—have been developed to address different scales of analysis, from process-level performance to molecular-level interactions [179].

Empirical models, often based on experimental correlations, provide practical tools for predicting membrane performance. These models link permeability and selectivity to membrane properties and operating conditions but are typically limited by the range of data from which they are derived [43,180,181,182]. Algebraic models, as used by Petterson and Lien, offer simplified representations of membrane modules, enabling the evaluation of performance in single- or multi-stage configurations under idealized conditions [183]. More advanced steady-state models for multicomponent gas separation have been proposed to improve accuracy and computational efficiency [184].

Recent efforts have incorporated machine learning (ML) to extend the predictive capabilities of empirical models. Explainable ML approaches have been successfully applied to correlate polymer structure with gas separation performance, allowing for the design of novel high-performance materials [185]. ML has also been employed in module design and process optimization, providing the rapid evaluation of complex input–output relationships [186,187,188].

Analytical model and the Maxwell–Stefan framework [189,190] enable the prediction of gas flux, selectivity, and permeability under defined conditions. These models capture key physical transport mechanisms and have been extended to include dual-mode sorption and non-ideal effects. Key contributions include Chern et al. [191] dual-mode sorption model for isothermal systems and Scholz et al. [192] inclusion of non-ideal effects like pressure drops, Joule–Thomson effects, and concentration polarization. Wang et al. [193] further enhanced modeling realism by incorporating flow dynamics and pressure variations in CO_2_/CH_4_ separation.

Computational approaches such as computational fluid dynamics (CFD) and finite element analysis (FEA) provide detailed insights into transport phenomena, flow distribution, and mechanical stability. CFD has been used to study shell-side flow, packing density, and flux distribution in hollow fiber modules [194,195,196]. FEA is particularly valuable for assessing stress distribution and deformation in hollow fibers under operational loads, such as high pressure and temperature [197]. Numerical methods, including finite difference, finite element, and orthogonal collocation, have been applied to simulate mass transport in complex module configurations [198,199,200]. These models are often integrated into process simulation platforms such as MATLAB or Aspen HYSYS for full-system analysis and optimization [201,202]. The formation of hollow fiber membranes via phase inversion has been studied using phase-field models, which simulate the evolution of membrane morphology based on polymer–solvent interactions and thermodynamic parameters. These models offer predictive capabilities for pore structure and skin layer development during fiber spinning [203,204,205].

Molecular dynamics (MD) simulations have become essential tools for investigating gas transport mechanisms at the nanoscale. While their application is limited to dense regions, such as dense flat membranes or the skin layer of hollow fibers, MD simulations provide detailed insights into how polymer chain architecture, free volume, and side group rigidity affect gas permeability and selectivity [206,207,208,209,210,211].

MD has also been applied to study advanced materials such as PIMs, TR polymers, and MMMs. These simulations allow for the prediction of key properties including plasticization resistance, aging behavior, and dynamic gas–polymer interactions [212,213,214,215,216,217].

In recent years, MD has been extended to model hybrid organic–inorganic materials and nanostructured systems with complex transport behavior [218,219,220,221,222].

Modeling efforts are also focusing on simulating more realistic operating conditions. Most current studies are based on pure gas models, whereas real industrial scenarios involve complex mixtures, often containing impurities such as NO_x_ and H_2_S [223]. Capturing the competitive sorption and transport dynamics of multicomponent systems is essential for the assessment [224,225]. Additionally, multiscale modeling approaches that integrate molecular simulations with module- and process-level models will be key to the next generation of hollow fiber membrane design.

## 5. Industrial Scalability of Hollow Fibers for Gas Separation

The industrial scalability of hollow fiber membranes for gas separation depends on several factors, outlined as follows: optimized manufacturing techniques, robust module design, precise control of process parameters, and a strong focus on both the cost-effectiveness and long-term performance.

### 5.1. Manufacturing Methods and Their Advantages

The most suitable manufacturing method for industrial-scale production varies with the type of fiber being produced. Wet spinning and dry-jet wet spinning, for instance, are the go-to industrial methods for creating polymeric hollow fiber membranes that have an external selective layer. Interestingly, while less common for gas separation membranes, melt spinning offers significant advantages. It boasts high production rates and avoids the use of solvents, which can be a big plus from both an environmental and cost perspective. Crossflow filtration and continuous coating methods are crucial for composite hollow fiber membranes (like thin-film composite or dual-layer types). These techniques enable the direct formation of selective hollow fibers while they are already assembled into the modules [107].

### 5.2. Cost–Benefit Considerations and Durability

The cost–benefit ratio for producing hollow fiber membranes for gas separation depends on several factors. Key among these are their durability and the trade-off between the material cost to produce them and the membrane’s efficiency in the separation process. The glassy polymers with rigid backbones and high glass transition temperatures (T_g_), offer better durability [226]. These include PIs, PSF, PES, and fluoropolymers. Conversely, polymers with lower durability (or susceptibility to specific issues) include the following: rubbery polymers (e.g., PDMS, Pebax) and polymers of intrinsic microporosity (PIMs) [227].

### 5.3. Balancing Durability with Performance: The Role of Robeson Plots

**Figure 11:**
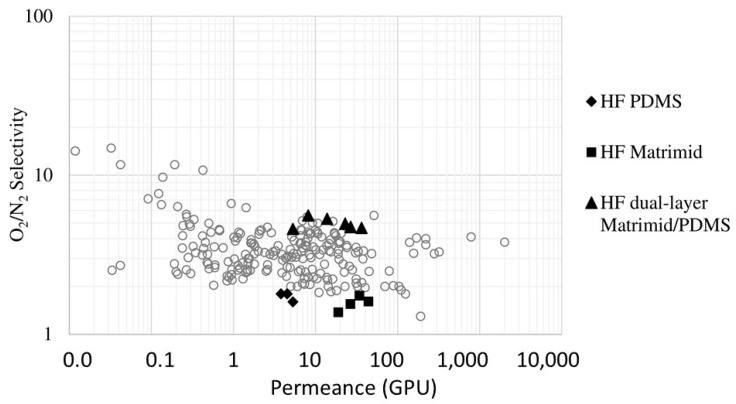
Example of typical Robeson plot for O_2_/N_2_ separation of different HF types of PDMS, Matrimid or Matrimid/PDMS dual layer membranes The open circles represent other membranes reported in the literature [19].

A thorough evaluation of membrane materials must extend beyond just durability to encompass performance metrics like permeability and selectivity [43]. For example, while PIMs are known to be affected by physical aging, they simultaneously exhibit unprecedented permeability, often surpassing other polymer types. This highlights a critical balance [58]. To assess the overall effectiveness of a membrane, particularly when considering new materials or fabrication techniques, Robeson plots are an invaluable tool. These plots provide a clear visual benchmark for evaluating the trade-off between permeability and selectivity, effectively guiding the development of next-generation materials with improved performance. While commonly used for flat sheet membranes, Robeson plots are equally applicable and crucial for hollow fiber membranes, serving as a fundamental guide for all those operating in the field of membrane engineering for gas separation [26]. An example of a Robeson plot for different types of hollow fibers used for O_2_/N_2_ separation is shown in Figure 11 below [19].

## 6. Hollow Fibers for Gas Separation Processes

Hollow fibers display unique properties, which make them highly attractive in many industrial gas separation processes. The use of hollow fiber membranes on an industrial scale is an effective technology for transforming traditional industries into the more sustainable ones. The use of HFMs in various gas separation processes, such as post-combustion carbon capture from flue gas (CO_2_/N_2_) [228], natural gas treatment [229], biogas up-grading (CO_2_/CH_4_) [131], oxygen-enriched air production (O_2_/N_2_) [36,230,231], and hydrogen recovery (H_2_/CO_2_) [232], could address some of the most significant challenges of our modern society. In some specific cases, they can even convert by-products into valuable new products, aligning with the increasingly necessary concept of a circular economy [233].

Their primary industrial gas separation applications of commercial hollow fiber are outlined in Table 4.

### 6.1. Helium Separation from Natural Gas

Recovering helium from natural gas using HF membrane separation is a viable alternative to traditional cryogenic distillation. Helium is extensively used in various scientific, medical, and industrial applications. In hospitals and analytical laboratories, it acts as a coolant for magnetic resonance imaging (MRI) and nuclear magnetic resonance (NMR) analysis. Helium also functions as a carrier gas in analytical and scientific equipment and as an inert gas in welding. Additionally, helium-oxygen mixtures are utilized in deep-sea diving for pressurizing and purging pressure vessels, and helium is integral to rocket technology [60]. The traditional and integrated cryogenic distillation process is followed by the separation of the crude helium stream using a hollow fiber system, and finally, helium upgrading occurs through pressure swing adsorption (PSA) [234]. The world’s first plant to recover high-purity helium from nitrogen-rich natural gas without the need for a cryogenic stage was built in Canada by Linde Engineering. This innovative facility exclusively employs an integrated system of PSA combined with Evonik’s SEPURAN^®^Noble hollow fiber membrane technology. SEPURAN^®^ modules elevate the crude gas to a helium content of approximately 50%. From this resulting gas mixture, nearly 90% pure helium is then extracted through the pressure swing adsorption process [234]. Although many commercial companies manufacture HF membranes for helium extraction from natural gas, there is a limited amount of research published on the use of HF membranes specifically for helium recovery. Häussinger et al. [241] developed an aromatic PI HF system with an effective area of 9000 m^2^ m^−3^, capable of recovering helium at 95% by means of several stages of HF membrane separation processes. Shoji and Moriya describe a helium purification method based on glassy polymer (polyolefin/cellulose/silicon) HF membranes. The glass HF walls have pores of 1.5 nm, which were created by the incorporation and the subsequent leaching of alkali metal ions in the wall surface. The selectivity of these HFs for helium over N_2_ is about 1800–2000 [242]. Dibrov et al. [50] presented an extensive study on Asymmetric Torlon^®^ HFs for helium separation from natural gas. The HFs were treated with PDMS in order to plug the defects in the thin dense skin layer. The module of 200 fibers with a selective layer of 82 nm showed an active area of 0.177 m^2^ with a helium permeance of about 0.1 m^3^(STP) m^−2^·h^−1^·bar^−1^) and a He/CH_4_ selectivity of 340 [50]. In conclusion, while cryogenic distillation remains a common method, the use of hollow fiber membrane separation, particularly when integrated with PSA technology, presents a compelling and increasingly viable alternative for efficient and high-purity helium recovery from natural gas, demonstrating significant advancements in both industrial application and ongoing research. Table 5 reports some examples of HFMs with their respective helium permeabilities and ideal He/CH_4_ selectivities.

### 6.2. H_2_-Recovery

The chemical process sector is experiencing a rising demand for hydrogen. As a readily storable and transportable energy source, H_2_ is the most promising future energy carrier. Since Monsanto’s development of PSf_HFMs in 1970, which were the first hollow fiber membranes implemented in a pilot plant for ammonia synthesis gas separation [247], other companies have specialized in hollow fiber membrane systems capable of high-purity hydrogen separation. H_2_ can be obtained by the purification of gas mixtures coming from industrial processes such as ammonia purge gas recovery, oil refinery applications, CO purification, methanol purge gas recovery, and petrochemical applications. In all cases, to increase the power output of hydrogen fuel, CO_2_ must be removed. The membranes used for this separation can be categorized into two categories as follows: hydrogen-selective membranes and carbon dioxide-selective membranes. As a rule, hydrogen-selective membranes are suitable for moderate high hydrogen recovery with high hydrogen purity at low pressure. In contrast, carbon dioxide-selective membranes are preferred for achieving moderate hydrogen purity with high hydrogen recovery at high pressure [248].

#### 6.2.1. H_2_-Selective Hollow Fiber Membranes

H_2_-selective HF membranes work by molecular sieving mechanisms. This separation mechanism is particularly effective at separating H_2_ from carbon dioxide (CO_2_). Kumbharkar [249] and their team were the first to create asymmetric HF membranes made of PBI that can separate H_2_ and CO_2_ at high temperatures. These PBI fiber membranes significantly improved the rate of hydrogen gas permeation (P_H2_) by up to eight times, reaching a value of 2.6 × 10^−6^ cm^3^(STP) cm^−2^·s^−1^·cmHg^−1^. Additionally, these membranes exhibited a selectivity for H_2_ over CO_2_ of approximately 27 at 400 °C, which was significantly higher than their performance at 100 °C. The excellent separation performance at elevated temperatures indicates the potential use of these hollow fibers for high-temperature hydrogen separation applications. Additionally, hydrogen separation from carbon monoxide was performed using a PI hollow fiber membrane module manufactured by Peer’s group [250]. Extracting hydrogen from gas mixtures containing CO_2_ requires multiple steps. Polymeric membranes can be very effective for separating gas pairs such as H_2_/CO, H_2_/N_2_, or H_2_/CH_4_, which have significantly different molecular sizes. This is because glassy polymers like PSf, CA, and PI are preferred over rubbery polymers for the development of HFMs. For example, Favvas et al. [251] synthesized Matrimid^®^5218 HFs, characterized by a hydrogen permeance spanning 20–52 GPU across a temperature window of 40–100 °C. These membranes exhibited superior separation performance, achieving a maximum H_2_/CO_2_ selectivity of 37.8 and an optimal H_2_/CH_4_ selectivity of 137 at 40 °C and 60 °C, respectively. The recent years have seen the exploration of PIM-EA(Me_2_)-TB a PIM containing Tröger’s base units in the chain, for the creation of highly H_2_-selective HFs. While PIMs are generally CO_2_-selective, PIM-EA(Me_2_)-TB [56] exhibits a strong size-sieving character. It boasts a high H_2_ permeability of 7760 Barrer and demonstrates significant selectivity for H_2_ over O_2_, CH_4_, and N_2_, with separation factors of 7, 11.1, and 14.8, respectively [252]. Bernardo et al. [253] reported thin-film composite high-flux membranes based on the closely related PIM-EA(H_2_)-TB polymer. These membranes exhibited H_2_/N_2_ selectivities of up to 38, depending on the precise coating parameters. An additional strategy for increasing the size selectivity of polymeric HFs and particularly the H_2_/CO_2_ selectivity is the introduction of porous fillers possessing well-defined pore sizes such as the created ZIF-7 membranes on PVDF_HF supports [254]. These membranes showed impressive hydrogen gas permeability of approximately 7000 GPU, along with a high selectivity for hydrogen over carbon dioxide of 18.4 at a pressure of 1 bar and a temperature of 298 K. At 550 °C and 2 V voltage, a hydrogen flux of 2.15 mL·min^−1^·cm^−2^ and a hydrogen recovery rate of 81% are obtained when the feed is a low-concentration (13 vol.%) H_2_ stream. A novel PBI-based HFM synthesized by Singh et al. [255], utilizing a unique dope composition incorporating an acetonitrile diluent and a water coagulant, has demonstrated superior gas transport performance. This innovative fabrication technique yielded asymmetric, macro-void-free HFMs. Through the careful evaluation of the influence of the dope viscosity, the coagulant chemistry, and the air gap on the HFM morphology, the researchers achieved impressive high-temperature (up to 350 °C) H_2_ permeances of 400 GPU, with H_2_/CO_2_ selectivities exceeding 20. This represents a significant advancement in gas separation technology [256]. Wang et al. [257] obtained a biomass-derived CHFMs with ultrahigh H_2_/CH_4_ separation factor of 4149 under a simulated Hydrogen Natural Gas (H2NG) (10 mol% H_2_/90 mol% CH_4_) at 30 bar [257]. To evaluate the feasibility of the CHFMs for H_2_ extraction from H2NG, a three-stage membrane system was designed based on the mixed gas separation performances. It suggested that 99.99 mol% H_2_ can be achieved with 90% recovery, and the specific H_2_ purification cost was 0.245 $ Nm^−3^ for a 1.8 × 10^5^ Nm^3^ h^−1^ production scale, which provides the possibility of hydrogen extraction from pipelines [257]. Table 6 shows some examples of HFMs with their respective H_2_ permeances and H_2_/CO_2_ and H_2_/CH_4_ selectivities.

#### 6.2.2. CO_2_ Selective HFMs

CO_2_-selective HF membranes, often referred to as reverse-selective HF membranes, are typically constructed from rubbery polymers that have a strong affinity for CO_2_ molecules. This affinity leads to a gas transport mechanism primarily driven by solubility selectivity [254]. Polymers like PEO, PEO-based crosslinked copolymers (e.g., PEO-PI), PDMS, and Pebax^®^ have demonstrated promising performance in terms of balancing CO_2_ permeability and CO_2_/H_2_ selectivity. However, their direct spinning is challenging, limiting their application to composite HF membranes for CO_2_/H_2_ separation. In 2014, Chen et al. [48] pioneeringly obtained composite HFs for CO_2_/H_2_ separation using Pebax^®^/PDMS/PAN. These membranes exhibited impressive gas separation performance, with a CO_2_ permeance of 481.5 GPU and a CO_2_/H_2_ selectivity of 8.1 [48]. Subsequently, Hu’s group further enhanced the CO_2_/H_2_ solubility selectivity of Pebax^®^ 1657 composite HFs to 22 by incorporating SAPO-34-NH_2_ and a CO_2_-philic ionic liquid [263]. SAPO-34/PDMS HF mixed-matrix membranes have also been explored for the emerging biological hydrogen production process, where CO_2_/H_2_ separation can be carried out at lower temperatures. These membranes achieved CO_2_ and H_2_ permeability coefficients of 3285 and 569 Barrer, respectively, with a maximum CO_2_/H_2_ selectivity of 6.1 at 120 kPa feed pressure [264]. Another strategy was adopted by Wang et al. [265] who developed a cellulose-based CMS_HFMs using a post-treatment strategy involving hydrogen reduction and oxygen doping (H-O treatment) [265]. This treatment significantly improves the CO_2_ affinity of the membrane. The hydrogen reduction process, carried out at temperatures between 500 and 600 °C, creates active sites within the carbon structure. The subsequent oxygen doping step introduces oxygen-containing functional groups, which enhance both CO_2_ adsorption and diffusion rates. These modifications make the membrane highly effective for purifying natural gas under high-pressure conditions [265].

Yong et al. created hollow fiber membranes composed of PIM-1 and Matrimid^®^, which exhibit enhanced separation capabilities [58]. By incorporating 5% and 10% PIM-1 into the fibers, the CO_2_ permeance increased by 78% and 146%, respectively, without negatively affecting the CO_2_/CH_4_ selectivity compared to pure Matrimid^®^. Further increasing the PIM-1 content to 15% and applying a silicone coating resulted in a significant boost in CO_2_ permeance to 243.2 GPU, along with a CO_2_/CH_4_ selectivity of 34.3. When tested with a 50/50 CO_2_/CH_4_ gas mixture, this fiber demonstrated a CO_2_ permeance of 188.9 GPU and a CO_2_/CH_4_ selectivity of 28.8 [58].

### 6.3. O_2_/N_2_ Separation

The membrane-based separation of oxygen and nitrogen represents a major technological advancement with far-reaching consequences for diverse industries. Nitrogen, a highly valuable resource, plays a crucial role in numerous applications. It functions as an inert gas, a coolant, a combustion modifier, an ingredient in adhesives, and a raw material for chemical and plastic production [266]. Pure oxygen finds application in a diverse array of industrial sectors, such as healthcare, food processing, farming, petroleum refining, and electricity production [267]. Hollow fiber membrane-based gas separation is rapidly advancing, offering efficient solutions for producing nitrogen and oxygen-enriched air for a variety of applications. Some applications could be in food packaging (Modified Atmosphere Packaging—MAP) [268], electronics manufacturing (preventing oxidation), laser cutting [269], and as an inert gas blanket in chemical and pharmaceutical processes [270]. On-site nitrogen generation using membranes offers a cost-effective and reliable alternative to traditional cryogenic distillation or delivered liquid nitrogen. Oxygen enrichment of air is a crucial process to obtain oxygen-rich streams for medical and numerous industrial applications [151]. In recent years, combustion with oxygen-enriched air (OEA) is seen as a good method to facilitate energy-efficient carbon capture from power plants because it eliminates the presence of nitrogen in the flue gas. Hence, the flue gas is mainly composed of CO_2_ and condensable water that can be easily separated [271]. Using OEA is an important strategy for reducing processing costs and CO_2_ emissions in several industrial processes. Matson et al. [272] made the first assessment of a membrane process for OEA in 1986. They explored the use of polyphenylene oxide membranes with O_2_/N_2_ selectivity of 4.8 and O_2_ permeability of 17 Barrer, and they concluded that the membranes process can be profitable for the modest purity range (30–50% O_2_) and small-scale industrial plans [272]. Kimura and Browall proved the viability of a membrane-based oxygen enrichment process for direct-flame heating systems by achieving an oxygen purity of 33% [273]. High-purity OEA (90–95% O_2_) can be employed in oxy-combustion processes to capture or utilize the concentrated CO_2_ from flue gas. A high of oxygen purity was obtained by Chuah et al. [274], who developed an asymmetric, defect-free hollow fiber membrane composed of Matrimid^®^ 5218 polyimide for large-scale production in 2-inch membrane modules. These modules can achieve an effective surface area of up to 2.6 square meters and a packing density of up to 44%, enabling the production of oxygen-enriched gas (OEG) from simulated air with an oxygen purity of 45% [274].

### 6.4. H_2_O/Air

**Figure 12:**
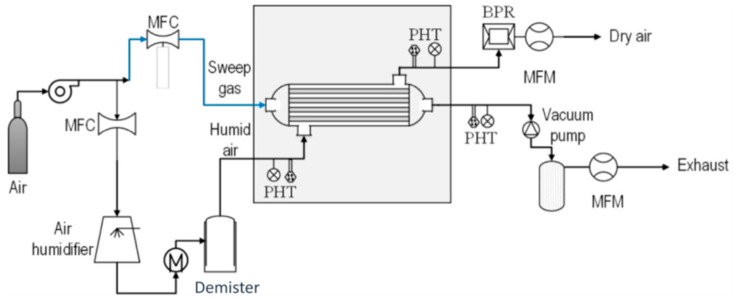
Schematic representation of dehumidification setup [281] with permission from Elsevier.

Air dehydration is the process of removing moisture from air. This is also known as air dehumidification or air-drying. Because air naturally contains moisture, air dehydration is a widely used membrane technology. There are two main methods for membrane air dehydration as follows: the sweep gas method and vacuum method. The sweep gas method is based on the diffusion of water vapor through the membrane and then it is carried away by a dry sweep gas. On the other hand, in the vacuum method the water vapor diffuses through the membrane and is pulled away by a vacuum. Even though the moisture level in the sweep air might be similar to that in the compressed air, the higher pressure of the compressed air creates a significant difference in water vapor partial pressure. This pressure difference allows the water vapor to move from the high-pressure compressed air to the low-pressure sweep air through a tailored membrane. Hollow fiber membrane technology has thus far also been applied for the removal of water vapor from compressed feed gas or air streams [275]. Zhao et al. [276] investigated the performance of hollow fiber membranes for air dehumidification at one atmosphere. Nine one-inch PAN/PDMS hollow fiber membrane modules were used to create a pilot-scale air dehumidification system. A 150 h test, using a low lumen-side vacuum of 0.78 bar absolute pressure, showed good water vapor transport. The system reduced the water vapor concentration of the humid air feed from 18–22 g/m^3^ to 13.5–18.3 g/m^3^, achieving energy savings of up to 26.2% compared to traditional air conditioning [276]. The dehumidification modules employ multiple assemblies of hollow fiber membranes. Humid air is introduced to the shell side at ambient pressure, whereas the lumen side of the fibers is subjected to a vacuum. These membranes are often employed to dry air compressed to around 10 bar [277]. This air-sweep dehumidification technique is also utilized in laboratory settings, where Nafion membrane tubes are used to facilitate moisture exchange [278]. Liu et al. [279] investigated two different ways of poly(dopamine) PDA modification for increasing the separation performance in terms of water vapor permeance and the water vapor/H_2_ selectivity. They found that the surface-modified PVA/PVDF hollow fiber composite membranes with PDA resulted in a dense, thin layer with high nitrogen solubility, negatively impacting water vapor removal efficiency [279]. In contrast, the three-layer PDA_PVA_PVDF substantially improved dehumidification capability. The effects of modification conditions (PDA concentration and time) on the water vapor permeance and water vapor/H_2_ selectivity of the PDA_PVA_PVDF membrane were examined. The maximum water vapor permeance of 2898 GPU was observed when using a 0.1 g L^−1^ PDA solution and a 30 min modification time. Research has indicated that surface modification with PDA is a key factor in improving the hydrophilicity and water vapor/H_2_ separation capabilities of PVA/PVDF composite membranes [279]. For the first time, Ingole et al. [280] proposed the integration of MOFs into thin-film nanocomposite (TFN) hollow fiber membranes for enhancing the water vapor permeation from gaseous mixtures. Hollow fiber membranes composed of PSf were employed as a base material for applying a thin nanocomposite coating. Interfacial polymerization (IP) was carried out using m-phenylene diamine (MPD) and trimesoyl chloride (TMC) as monomer solutions. The incorporation of a small amount of MOF particles significantly enhances water vapor transport through the resulting TFN membranes. The water vapor permeance was increased from 785 GPU, for TFN membrane, to 2244 GPU (MOF@TFN3) [280]. Upadhyaya et al. proposed as alternative to TFC hollow fiber membrane porous Ultem^®^ hollow fibers coated with a thin layer of green tannic acid, a plant-based polyphenol derived from sources like oak bark and tea leaves. The membrane selectivity was improved by chemically bonding (crosslinking) the selective layer with MPD or hyperbranched PEI in water. Semi-industrial membrane modules, containing 350–500 hollow fibers, each 30 cm long, were produced. Long-term continuous operation of these modules (over a year) showed increased dehumidification performance and an excellent Coefficient of performance COP (Figure 12) [281]. The same scientists propose applying a layer of NEXARTM, a commercially available pentablock copolymer, onto polyetherimide hollow fiber supports. This method aims to separate water vapor from moist air. As the block copolymer solution undergoes a structural change, it forms a distinctive layered structure with alternating lamellar and parallel cylindrical phases. This unique structure achieves a water vapor permeance of up to 9089 GPU and a water vapor to nitrogen selectivity of up to 3870. It demonstrated the capability to reduce relative humidity from 80% to 41%, making it a promising option for membrane dehumidification applications [282].

### 6.5. CO_2_ Separation Processes

#### 6.5.1. Biogas Upgrading and CO_2_/CH_4_ Separation

**Figure 13:**
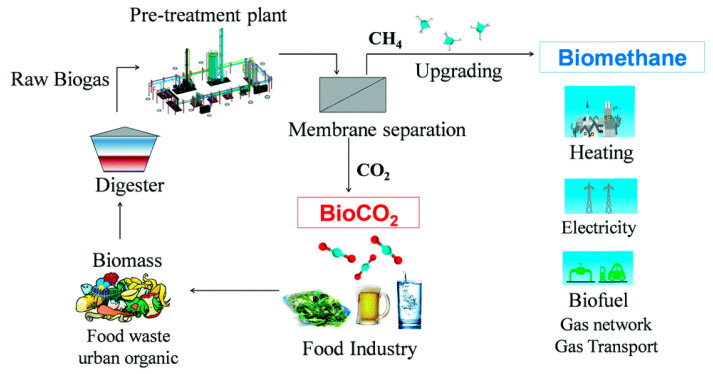
Schematic representation of the sustainable cycle of anaerobic digestion with simultaneous production of biomethane and food grade BioCO_2_. Reprinted from [131].

Polymeric gas separation membranes are playing an increasingly important role in biogas upgrading [283] from large-scale industrial applications processing organic waste [131] to thousands of smaller units operating globally [284]. Hollow fiber membrane-based technologies provide a sustainable and low-maintenance solution for biogas upgrading. They eliminate the need for chemical absorbents, such as amines, which can have detrimental environmental impacts [285]. Recent advancements in biogas upgrading using membrane separation technologies primarily focus on optimizing the process configuration, exploring new membrane materials, and integrating established membrane-based processes with biogas upgrading techniques [286]. Esposito et al. [131] carried out a case study on the feasibility of biogas upgrading at full industrial scale to distribution grid quality methane, and simultaneous recovery of food-grade CO_2_, starting from organic waste (Figure 13).

Nowadays, Evonik’s SEPURAN^®^ Green PI membranes are the most used HF membranes in biogas upgrading processes [287]. These hollow fiber membranes are used in a three-stage separation process in one of Europe’s largest industrial biogas-upgrading plants located in Italy (Montello/Tecno Project plant) that has a capacity to treat 15,000 Nm^3^ h^−1^ with a biomethane production capacity of 9000 Nm^3^ h^−1^ and simultaneous recovery of 7000 tons of CO_2_ per year. The membranes used in the plant are approximately 1.3 m long, containing several tens of thousands of hollow fibers, each with a diameter of 0.5 mm, and for improved process efficiency, these membranes work at a temperature of 50 °C and a pressure of 17 bar. From this study it emerged that the resulting biomethane, exceeding 96% purity by volume, reaches the purity needed for injection into the natural gas grid. This is one of the first successful industrial plants where there is a simultaneous production of renewable energy as biomethane and CO_2_ reuse, while it was traditionally discharged as waste. Building on the success of industrial applications like the Montello plant, researchers are continuously exploring optimal membrane configurations and operating conditions to further enhance biogas upgrading efficiency.

Shin et al. [288] studied different number and type of membrane stage for biogas upgrading in which modules could be set up in different ways, either as a single-stage, two-stage, or three-stage system. They found that the use of multiple stages is the most effective way to achieve high CO_2_ concentration, maximize CO_2_ recovery, and minimize methane loss. However, as the number of stages increases, optimizing the process becomes more challenging. This is where computer simulations can be helpful in designing the process or in evaluating a limited number of configurations under specific conditions. In addition to the membrane configuration, factors like temperature and pressure can also impact the efficiency of biogas upgrading. Researchers have extensively studied these factors to improve the overall process. For example, Abejon et al. [289] investigated the economic feasibility of multi-stage CO_2_/CH_4_ separation processes. They employed novel hollow fiber membranes, such as modified PDMS and ionic liquid-chitosan composites. Case studies focused on biogas upgrading, natural gas purification, and enhanced oil recovery. Results suggested that large-scale implementations of these processes could be cost-competitive, with total costs potentially less than 0.050 USD per m^3^(STP) of treated gas. Xe et al. [290] evaluated cellulose-based hollow fiber carbon membranes for separating CO_2_ and CH_4_ at moderate pressures (5–20 bar), achieving a CO_2_/CH_4_ permselectivity exceeding 60. Their findings suggest the following: (a) carbon membranes could be a cost-effective solution for biogas upgrading at a feed pressure of 8.5 bar, (b) enhanced membrane performance could further decrease costs, and (c) carbon membrane systems could be particularly economical for small-scale biogas upgrading plants with capacities around 350 m^3^(STP) h^−1^. Brunetti et al. [291] presented cellulose-based carbon hollow fiber membranes exposed to a gas stream containing H_2_S and water vapor. Before the long-term tests, the membranes were characterized for their CO_2_ and CH_4_ sorption (2.98 and 2.00 mmol g^−1^, respectively, at 298 K and 10 bar), diffusion (2.45 × 10^−7^ cm^2^ s^−1^ for CO_2_), and permeation properties (120.9 and 2.3 Barrer for CO_2_ and CH_4_, respectively, at 308 K) using single gases. Typical HF membranes used in natural gas treatment are reported in Table 7.

#### 6.5.2. CO_2_/N_2_ and Flue Gas Purification

**Figure 14:**
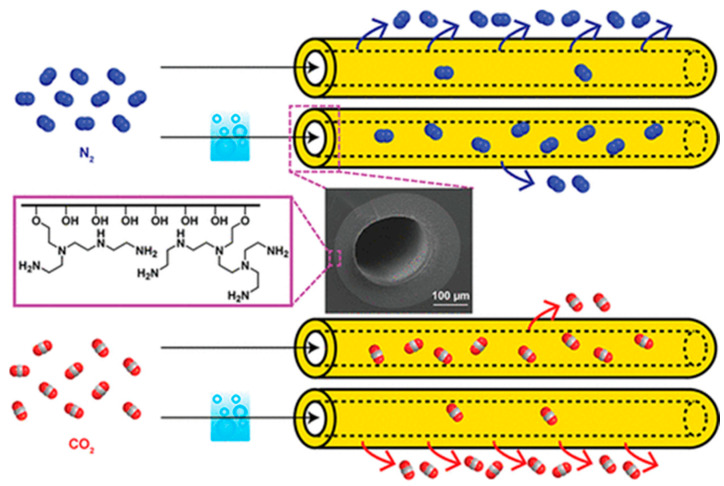
Graphical representation of N_2_—or CO_2_ selective membranes [296], reprinted with permission from ACS.

CO_2_/N_2_ separation is crucial for purifying flue gas, the waste product of fossil fuel combustion. Post-combustion CO_2_ capture, occurring at relatively low pressures and temperatures, targets exhaust streams rich in nitrogen (approximately 80 vol.%) but containing only 5–25 vol.% CO_2_, along with trace pollutants like CO, NO_2_, and SO_2_. Hollow fiber membranes offer a promising approach to capturing this CO_2_ and mitigating current emission challenges. Kim et al. [296] proposed a hollow fiber-supported mesoporous silica membranes amine-functionalized with aziridine to yield hyperbranched aminopolymers within the membrane pores. Surprisingly, the modified membrane exhibits a preference for nitrogen gas over carbon dioxide when operated in dry conditions (Figure 14). The study reveals the effects of strong adsorption of CO_2_ under dry permeation conditions, leading to reduced CO_2_ diffusivity because of CO_2_-induced amine crosslinking in the mesopores. On the other hand, the hyperbranched aminosilica membrane shows CO_2_-selective properties under humid conditions. Water molecules cause a lower degree of amine crosslinking and thus facilitate the transport of CO_2_.

CO_2_-selective membrane technology offers distinct advantages. By exploiting the inherently high N_2_ concentration in power plant flue gas, it enables the direct extraction of a high-purity CO_2_ stream from the high-pressure side of the membrane. This can result in significant energy savings associated with the final compression stage of the CO_2_ product prior to storage. A recent report investigated the feasibility of employing N_2_- and CO_2_-selective membrane technology for post-combustion CO_2_ capture in power generation (Table 8).

### 6.6. Olefin/Paraffin Separation

In spite of the drive towards decarbonization and the use of biopolymers and renewable resources, a scenario without a petrochemical industry that produces massive amounts of ethylene and propylene as the building blocks for polymers and chemicals is unimaginable. Olefin/paraffin separation is one of the key processes in the chemical industry, which, at least for two fundamental reasons, has surpassed the production of other commodity organic compounds [303]. First, ethylene and propylene are key feedstocks for producing polyethylene and polypropylene, two of the most important commercial polymers; and second, this is due to the role of these chemicals as starting materials [304]. Nevertheless, the nearly identical physical properties of olefins and paraffins make this separation highly challenging. Traditionally, olefin/paraffin separation is performed by cost- and energy-intensive cryogenic distillation and given their enormous production volumes, the ethylene/ethane and propylene/propane separation by more efficient processes is highly desirable. The potential use of membrane processes is therefore a topic of great interest [305].

#### 6.6.1. Polymeric Membranes

One promising solution to this issue is employing non-selective microporous hollow fiber membranes that exploit the potential of capillary condensation. These membranes present unique advantages such as possessing an exceptionally high surface area-to-volume ratio [306] and the ability to remain unaffected by the complexities of two-phase fluid mechanics. Most importantly, hollow fibers can enhance heat and mass transfers via functioning as a phase contactor [307,308]. The first introductory work on utilizing the capillary condensation mechanism for separating light gases within porous materials resulted in a U.S. patent by Calamur et al. [309]. Based on this breakthrough, further research explored the possibility of replacing traditional distillation columns with structured packing media such as non-selective membranes, for water-isopropanol and light hydrocarbon mixture separations [310,311]. Using this concept to propene/propane distillation, Yang et al. [312] reported at least ten times faster mass transfer rates in hollow fiber modules than those achieved in conventional tray towers. In addition, thermal stability studies [313] suggested PVDF, PS, polypropylene (PP), and mixed ester (ME) as stable polymers in olefin/paraffin medium below 100 °C. Subsequent research by the same group [314] revealed the influence of hollow fiber morphology and polymer compatibility on C_3_ separation efficiency and operational stability. Significant swelling of the fibers increased their length and surface area but introduced some challenges such as channelings, which could compromise the separation process. These findings highlighted the importance of balancing structural design along with the materials’ properties to achieve optimal performance in industrial applications.

#### 6.6.2. Hybrid Supported Membranes

Focusing on separation by molecular size, Kim et al. [315], studied the permeation of propane and propene in thin CMS membranes formed on 10 cm long alumina hollow fiber supports, at 25 °C and pressures up to 0.7 MPa (100 psi). The study reported a propene permeance of 72 GPU versus very low permeance of C_3_H_8_ (4.39 GPU), despite their almost similar physical properties and smaller kinetic diameter of propane (i.e., C_3_H_6_: 4.50 Å; C_3_H_8_: 4.30 Å), resulting in C_3_H_6_/C_3_H_8_ selectivity of 16.5. This phenomenon was attributed to the strong adsorption affinity of the CMS layer towards propene, which, interestingly, exhibited comparable initial permeance with that of the small gas molecules (i.e., Ar, N_2_, and CH_4_).

The employment of MOFs as a highly potential material to develop a new class of porous materials for gas separation has gained noticeable attention during the last decades [316]. In this context, interfacial microfluidic membrane processing (IMMP) is a novel technique, introduced to fabricate MOF-film membranes on polymeric hollow fibers, functioning based on a two-solvent interfacial concept to precisely control membrane formation on both the inner and outer surfaces of the fibers. This technique was first reported by Nair et al. [317], who prepared a ZIF-8 membrane with an average thickness of 8.8 μm, successfully deposited on the inner surface of PAI_HFs, with a pore size of 100 nm. The results of single fiber showed a propene permeance of 48 GPU and a mixed C_3_H_6_/C_3_H_8_ ideal selectivity of 12 [317]. This technique has the advantage that it can use low-cost polymer hollow fibers as supports, functioning at ambient temperature. It can achieve high gas permeance and, exploiting the molecular sieving effect of ZIF-8 membranes, also high selectivity, for instance a selectivity of 370 for the H_2_/C_3_H_8_ gas pair. This makes IMMP a promising method for the scalable production of MOF-based hollow fiber composite membranes. Further efforts on optimizing the IMMP operational conditions resulted in better control of the fiber microstructures made of selective ZIF-8 membranes on PAI hollow fibers [318]. The C_3_H_6_/C_3_H_8_ ideal selectivity increased to 180 under standard conditions, 1 bar and 25 °C, which still remained high under elevated temperature, 60 at 120 °C, and pressure, 90 at 9.5 bar, emphasizing the effectiveness and scalability of this strategy. Subsequent studies by the same group reported the development of ZIF-90/carbon membranes via a novel technique, combining fluidic membrane processing with chemically inert carbon hollow fibers [319]. Therefore, the pyrolytic conversion of crosslinked polymer hollow fibers was employed, which facilitates the application of a wide range of relatively harsh fluidic processing solvents and conditions. The fabricated membrane was utilized to separate butane isomers.

High propene/propane separation factors of ~55 and 108 were obtained with MOF-based membranes [320] produced by in situ MOF growth through the counter-diffusion concept to yield robust, defect-free ZIF-8 [320] and ZIF-67 [321] membranes, respectively. In this method metal and organic ligand precursors flow on either side of a porous support, facilitating the MOF layer to form at the interface. Thus, the polymeric membrane acts as the support for the selective MOF film. Li et al. [322] reported the fabrication of defect-free MOF-film hollow fiber membranes, via gel–vapor deposition, combining sol–gel coating with vapor deposition. The resulting MOF membranes possessed a very low uniform thickness, ~17 nm, and showed high C_3_H_6_/C_3_H_8_ ideal selectivity of 70. Scaling-up the technique to 30 polymeric hollow fiber supports with membrane area of 340 cm^2^ exhibited very promising results without selectivity reduction.

#### 6.6.3. Hybrid Mixed-Matrix Membranes

Poor scalability is one of the main barriers to the commercialization of inorganic membranes, which has been mitigated after introducing the concept of MMMs. This minimizes the required quantities of MOFs as filler particles within a polymeric matrix and makes the membrane cost-effective and scalable. Although most of MOF-based MMMs remained at lab-scale studies, some efforts have been reported on developing asymmetric hollow fiber MMMs, which, from a packing efficiency point of view, is one of the best practical membrane geometries [79,113,323]. As an example, Koros’s research group [314] produced dual-layer ZIF-8/6FDA-DAM mixed-matrix hollow fiber membranes by utilizing the conventional dry-jet/wet-quench fiber-spinning technique. ZIF-8/6FDA-DAM mixed-matrix hollow fiber membranes with 30 wt.% ZIF-8 NP loadings exhibited enhanced C_3_H_6_/C_3_H_8_ selectivity of almost 30. This marked an advancement in addressing the scalability requirement, moving from laboratory-scale innovations into practical, industrially relevant membrane technologies. In situ growth of ZIF-8 onto existing asymmetric 6FDA-DAM polyimide hollow fiber membrane by Park and Jeong [177] proved to be a highly successful method to produce membranes with a stable propene/propane separation performance (up to 25 days) and a promising separation performance with a C_3_H_6_/C_3_H_8_ separation factor of about 23 at a propene permeance of 2.15 GPU.

## 7. Future Outlooks and Emerging Trends in Hollow Fiber Membranes

The field of hollow fiber membranes (HFMs) is rapidly evolving, driven by the increasing demand for efficient, sustainable, and cost-effective separation technologies. Beyond the continuous refinement of existing materials and fabrication techniques, several transformative trends are poised to revolutionize membrane design, operation, and application [324].

One of the most promising frontiers is the use of artificial intelligence for assisting HF membrane design and optimization [324]. Traditional membrane development is often a laborious, trial-and-error process. Artificial intelligence, such as machine learning algorithms, can significantly accelerate this by predicting material performance based on structural parameters, processing conditions, and target separation requirements. AI models can analyze vast datasets identifying unprecedented selectivity, the permeability of a specific hollow fiber, and how to prepare it in order to tailor the desirable membrane [325].

Recognizing that no single separation technology is optimal for all scenarios, another significant trend is the development of hybrid separation systems, in which the hollow fiber membrane unit for gas separation works in synergy with other processes. Researchers are increasingly exploring the synergistic integration of HFMs for gas separation with other separation processes. An example is the combination of membrane gas separation with adsorption, distillation, or absorption that can boost the advantage of each method, mitigating individual limitations. The integration of a hollow fiber unit for gas separation could involve a pre-treatment step to concentrate the target gas, followed by a more selective or energy-intensive process for final purification.

Finally, the critical global challenge of climate change is propelling the integration of HFMs into carbon capture and utilization strategies. Hollow fiber membranes are particularly attractive for CO_2_ capture due to their high surface area-to-volume ratio, compact design, and potential for energy-efficient operation compared to traditional methods. Emerging trends involve not only more selective and permeable membrane materials for CO_2_/N_2_ separation from flue gas but also their integration into advanced CO_2_ conversion processes for converting CO_2_ into valuable products [326].

## 8. Conclusions

Gas separation is a fundamental technique in many industrial processes, with major consequences for energy efficiency and environmental sustainability. Its importance lies in the essential requirement to isolate specific gases from blends, a need found in sectors like petrochemicals, natural gas, power generation, and pollution control. Understanding and optimizing these separation methods is not just about operational effectiveness; it is becoming a crucial ethical and economic obligation in our move towards a more sustainable industrial future. Membrane technology has become a highly attractive and increasingly common alternative to traditional industrial gas separation methods. While cryogenic distillation, absorption, and adsorption have long been the standard processes for separating gas mixtures, membrane-based separation offers a compelling array of advantages, making it a more efficient, cost-effective, and environmentally sound approach for a growing number of applications. Superior selectivity is another key benefit of membrane technology. By carefully choosing the membrane material and pore size, high separation factors for specific gas pairs can be reached. This often leads to purer products and less downstream processing. Although conventional methods can also be selective, membrane technology allows for precise adjustments to separation properties at the material level, enabling tailored solutions for various gas mixtures. This review focuses on HFMs, now a primary technology in gas separation due to their high surface area-to-volume ratio that allows for compact systems and improved efficiency. It compares a range of fabrication methods for different types of hollow fiber membranes, as well as their characteristics and achieved gas separation performance. The composition of the dope solution is a critical design parameter in the fabrication of high-performance HFMs for gas separation. A thorough understanding of the complex relationships between the polymer, solvent, additives, and the resulting membrane morphology and properties is essential for tailoring HFMs to specific separation applications and achieving optimal performance in terms of permeability, selectivity, and stability. The careful manipulation of the dope composition allows for the creation of membranes with precisely engineered structures and functionalities, paving the way for more efficient and cost-effective gas separation processes. The thin selective skin minimizes resistance to gas flow, leading to high flux, while the porous substructure provides mechanical support, reducing the overall pressure drop across the membrane. This architecture has proven particularly effective in various gas separation applications, including nitrogen generation, hydrogen recovery, carbon dioxide capture, and natural gas purification. The gas separation performance of asymmetric HFMs is primarily governed by the properties of the selective layer material and its thickness. Polymers with high intrinsic selectivity and permeability are often employed to form this layer. The separation mechanism relies on differences in the diffusion and solubility of different gas species within the polymer matrix. Consequently, the selectivity and permeability of the membrane are inherently linked and often exhibit a trade-off, as described by the Robeson upper bound.

Despite the significant progress in HFM technology, there remains a critical need for further research to overcome existing limitations and develop next-generation membranes with enhanced performance. This necessity stems from the ever-increasing demands for more energy-efficient and environmentally sustainable separation processes in various industrial applications, including natural gas purification, carbon capture, hydrogen recovery, and air separation. Several key areas warrant intensified research efforts, like the development of high-performance materials; in fact, exploring novel polymeric materials with intrinsically superior separation properties is crucial. This includes investigating polymers with tailored chemical functionalities, enhanced thermal and chemical stability, and the ability to form ultra-thin, defect-free selective layers. Another important point is the optimization of spinning parameters; the fabrication of asymmetric HFMs involves a complex interplay of spinning parameters, such as dope composition, extrusion temperature, bore fluid composition and flow rate, air gap distance, and coagulation bath conditions. A deeper understanding of how these parameters influence the final membrane morphology and separation performance is essential for achieving optimal structures. Advanced modeling and simulation techniques, coupled with systematic experimental studies, can provide valuable insights for process optimization and the production of defect-free membranes with tailored properties.

## Figures and Tables

**Table 1:** Examples of TFC-HFs fabricated by spinning.

Polymers	Top-Layer Thickness, [µm]	PCO_2_ [GPU]	Selectivity [-]	Ref.
Torlon^®^	0.41	0.84	CO_2_/N_2_ = 25	[110]
Matrimid^®^+PIM-1	0.07	243	CO_2_/N_2_ = 25	[58]
ODPA−TMPDA/DAT Copolyimide	0.27	39	CO_2_/N_2_ = 44	[111]
PES+Ag^+^	0.079	38	CO_2_/N_2_ = 53	[112]
Ultem^®^1010+PIM-1	0.10	49	CO_2_/N_2_ = 21	[113]
P84/PES-PEBA	n.a.	1.42	CO_2_/CH_4_ = 56.5	[114]
PDMS/PEI	n.a.	51	CO_2_/N_2_ = 21	[51]
TR-PBO	0.19	2500	CO_2_/N_2_ = 16	[115]

**Table 2:** Examples of TFC-HFs fabricated by dip coating.

HF	Top Layer	Thickness [µm]	PCO_2_ [GPU]	Selectivity [-]	Ref.
PES	PEG	0.15	30	CO_2_/N_2_ = 50	[119]
PAN	Pebax^®^1657	0.5	59	CO_2_/CH_4_ = 12	[49]
PVDF/PTMSP	Pebax^®^1657	1	111	CO_2_/N_2_ = 92	[120]
PVDF	Pebax^®^1657/[EmimBF_4_]	4–5	306	CO_2_/N_2_ = 36	[47]
PSF	GO/Pebax^®^1657	0.3	28.08	CO_2_/N_2_ = 43	[83]
PES	PES/Silicone	0.04	(PO_2_ = 10)	O_2_/N_2_ = 6	[121]

**Table 3:** Alternative solvents with reduced toxicity or non-toxic properties for the preparation of hollow fiber membranes for gas separation.

Solvent	Chemical Structure	Polymer	Separation [α]	Ref
Triethyl phosphate (TEP)	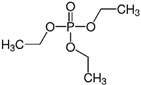	PVDF (as a support)	As a porous support	[122]
Gamma-butyrolactone (GBL)	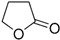	P84 (as a support)	CO_2_/CH_4_ = 26; 25 °C	[123]
Ethanol (EtOH)	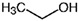	m-PBI (as a top layer)	H_2_/CO_2_ = 20.5; 150 °C 7 bar	[124]
EtOH/water (70/30 wt./wt.) mixture	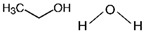	Pebax^®^1657 (as a top layer)	CO_2_/CH_4_ 18; 25 °C, 1–4 bar	[49]

**Table 4:** Commercial HF membranes used in industrial gas separation processes.

Separation	Application Field	Main Brands and Suppliers	
Helium separation	Helium purification	Sepuran^®^ Noble (Evonik)	[234]
H_2_ separation	H_2_ recovery	Sepuran^®^Noble (Evonik) HH, UBE H_2_, (Ube Industry)	[235]
		Polysep (UoP Honeywell company) PRISM^®^ (Air Products)	[236]
O_2_/N_2_	O_2_-enriched Air production Nitrogen generation	GENERON^®^ Sepuran^®^ N_2_ (Evonik) PRISM^®^ (Air Products) Ims (Praxair) Medal (Air Liquide) Aquilo (Parker Hannifin) (Ube Industry)	[237] [8]
H_2_O/air	Gas/liquid separation Air dehydration	PRISM^®^ (Air Products) Medal (Air Liquide) (Ube Industry) (Air Products) GENERON^®^	[8] [237]
CO_2_/CH_4_	Biogas up-grading Natural gas treatment	Sepuran^®^green (Evonik) psf-based membrane (Airrane Co., Ltd.) GENERON^®^	[131] [237]
CO_2_/N_2_	CO_2_ from flue gas	Polaris membranes (MTR)	[238]
CO_2_ separation	Acid gas treating Enhanced oil recovery Landfill gas upgrading	Cynara (Natco) Kvaerner (Grace Membrane System) (Ube Industry) (Air Products) GENERON^®^	[239] [237]
CO_2_/H_2_		Celazole (PBI performance products inc.)	[240]

**Table 5:** Helium permeability (GPU) and ideal He/CH_4_ selectivities of the relevant HFMs.

Polymer	Helium Permeability (GPU)	He/CH_4_	Ref.
Torlon^®^	7.4	370	[110]
P84	14.3	304	[243]
Ultem^®^	55	98	[79,244]
Matrimid^®^5218	195	12	[245]
6FDA-DAM-DABA	340	20	[161]
PBDI	50	100	[1]
PIM-PI/alumina precursor	40.0	11.2	[246]
PTMSP coated multi-layer TFC	5.96	36.39	[116]

**Table 6:** H_2_ permeance (GPU) and H_2_/CO_2_ and H_2_/CH_4_ selectivities in a range of different HFMs.

Polymer	H_2_ Permeance (GPU)	H_2_/CO_2_ (-)	H_2_/CH_4_ (-)	Ref.
PTMSP/Polyamide/PAN	7.79	28.3	40.1	[258]
PBI-sPPSU/PSf	16.7	9.7		[1]
PSf/TNT/PDMS	120		57.86	[16]
PBI/ZIF-8/PDMS	107	18.0	-	[259]
Polyaniline	5.0	7.9	-	[144]
Matrimid	66.05	5	-	[260]
sPPSU/PBI	16.7	9.7	-	[1]
TNTs/PSf	120		57.84	[16]
ZIF-8/PBI	107	18	-	[259]
GO/α-Al_2_O_3_	300	15	6.4	[261]
ZIF-8/Si_3_N_4_	2505	7.6	-	[262]

**Table 7:** Permeance and selectivity of HFs for CO_2_/CH_4_ and H_2_S/CH_4_ separation.

Polymer	CO_2_ [GPU]	CO_2_/CH_4_ [-]	H_2_S [GPU]	H_2_S/CH_4_ [-]	Ref.
Cellulose triacetate-HF	110	22	140	28	[292]
6FDA-based polyimides with bulky CF_3_ groups (PDMC−CF_3_)	35	32	32	30	[293]
Crosslinking-modified 6FDA-2,6-DAT-HFs	55	60	-	-	[160]
Co-polyimide grafted with β-Cyclodextrin-HFs	130	15	-	-	[294]
PDMC-ester-crosslinkable PI-HFs	120	30 *	-	-	[295] [172]

* Mix gas 50/50.

**Table 8:** HFs for CO_2_/N_2_—N_2_/CO_2_ separation.

Material for CO_2_/N_2_	Membrane	CO_2_ [GPU]		CO_2_/N_2_ [-]	T [°C]	Press. [bar]	Ref.
**PDMS/PAN**	PDMS/PAN composite HF membranes	3700		10	25	1	[129]
	PDMS/PAN composite-HF, PVP grafted	2500		12	-	-	[297]
**PEG**	PEG_HF-composite	30		50	25	2	[119]
**CA**	Cellulose acetate	13		39	25	3	[298]
**PSF**	PSF HF-commercialized by Airrane Co. (Daejeon, Republic of Korea)	120		26	25		[299]
	TPESU (new pes)	85		34	25	1	[171]
**PI**	Matrimid^®^	86		33	25	1	[58]
	P84^®^ (BTDA-TDI/MDI) co-polyimide	23		40	35	1	[300]
	6FDA-DAM-DABA	520		24	30	2.3	[301]
	BTDA-TDI/MDI co-PI (P84)	2.2		45–50	-	20	[243]
**PIMs**	PIM-1 composite multi-layer-HF Blend polymers	483		22			[118]
	PIM-1/Matrimid^®^	235		25	25	1	[58]
**Thermoplastic Polyolefin**	Poly(4-methyl-1-pentene) (PMP)	68.4		13.5	25	1	[302]
**Material for N_2_/CO_2_**	**Membrane**	**N_2_** **(GPU)**	**CO_2_** **(GPU)**	**N_2_/CO_2_** **[-]**	**T** **[°C]**	**Press. [bar]**	**Ref.**
**Mesoporous Silica**	hyperbranched aminosilica (HAS_dry)	12	1.8	6.7	35		[296]
	hyperbranched aminosilica (HAS_wet)	3.6	9.7	0.37	35		[296]

## Abbreviations

The following abbreviations are used in this manuscript:

ATRP	Atom transfer radical polymerization
CA	Cellulose acetate
CDA	Cellulose diacetate
CFD	Computational fluid dynamics
CH_4_	Methane
CHFMs	Carbon hollow fiber membranes
CMS	Carbon molecular sieve
CNTs	Carbon nanotubes
CO_2_	Carbone dioxide
COF	Covalent organic framework
COP	Coefficient of performance
CTA	Cellulose triacetate
DMSO	Dimethyl sulfoxide
ECMO	Extra-corporeal membrane oxygenation
EtOH	Ethanol
FEA	Finite element analysis
GBL	Gamma-butyrolactone
GO	Graphene oxide
GPU	Gas permeation unit
HF	Hollow fiber
HFMs	Hollow fiber membranes
ILs	Ionic liquids
IMMP	Interfacial microfluidic membrane processing
IP	Interfacial polymerization
MD	Molecular dynamics
ME	Mixed ester
ML	Machine learning
MMMs	Mixed-matrix membranes
MOF	Metal–organic framework
m-PBI	Meta-polybenzimidazole
MPD	M-phenylene diamine
MRI	Magnetic resonance imaging
N_2_	Nitrogen
NIPS	Nonsolvent-induced phase separation
NMR	Nuclear magnetic resonance
O_2_	Oxygen
OEA	Oxygen-enriched air
OEG	Oxygen-enriched gas
PAI	Polyamide-imide
PAN	Polyacrylonitrile
PBI	Polybenzimidazole
PDA	Poly(dopamine)
PDMS	Polydimethylsiloxane
Pebax^®^	Poly(ether-block-amide
PEG	Polyethylene glycol
PEI	Polyethylenimine, Polyetherimide
PES	Polyethersulfone
PFPs	Perfluoropolymers
PI	Polyimide
PIMs	Polymers of intrinsic microporosity
PLA	Poly(lactic acid)
PP	Polypropylene
PPO	Poly(phenyleneoxide
PSA	Pressure swing adsorption
PSF	Polyphenylsulfone
PS-PEGMA	Poly(styrene)-b-poly (ethylene glycol) methacrylate
PVC	Polyvinyl chloride
PVDF	Polyvinylidene fluoride
PVP	Polyvinylpyrrolidone
PTMSP	Poly[1-(trimethylsilyl)-1-propyne]
TEP	Triethyl phosphate
TFC	Thin-film composite
TFC-HFMs	Thin-film composite hollow fiber membranes
T_g_	Glass transition temperature
TIPS	Thermally induced phase separation
TFN	Thin-film nanocomposite
TMC	Trimesoyl chloride
TR	Thermally rearranged
STP	Standard temperature and pressure
VOCs	Volatile organic compounds
